# Galectin7 attenuates abdominal aortic aneurysm progression by resisting disturbed flow induced endothelial-to-mesenchymal transition

**DOI:** 10.7150/thno.117785

**Published:** 2026-01-08

**Authors:** Yanbing Wang, Yilin Zhou, Yeshen Zhang, Danni Tu, Guojun Chen, Yuan Han, Xiaomin Wei, Yanmei Chen, Senlin Huang, Yulin Liao, Wangjun Liao, Jiancheng Xiu, Yuegang Wang, Jianping Bin, Xinzhong Li

**Affiliations:** 1 Department of Cardiology, State Key Laboratory of Organ Failure Research, Nanfang Hospital, Southern Medical University, Guangzhou, China.; 2 Guangzhou Regenerative Medicine and Health Guangdong Laboratory, Guangzhou 510005, China.; 3 Guangdong Provincial Key Laboratory of Shock and Microcirculation, China.; 4 Cardiovascular Center, the Sixth Affiliated Hospital, School of Medicine, South China University of Technology, Foshan, China.; 5 Department of Cardiology, The Third Xiangya Hospital, Central South University, Changsha 410013, China.; 6 Cancer Center, the Sixth Affiliated Hospital, School of Medicine, South China University of Technology, Foshan, China.

**Keywords:** abdominal aortic aneurysm, disturbed flow, endothelial cells, endothelial-to-mesenchymal transition, galectin-7

## Abstract

**Background:** The switch to endothelial-to-mesenchymal transition (EndMT) in endothelial cells (ECs) induced by disturbed flow (d-flow) has been identified as the critical driver of the pathogenesis of inflammatory vascular disorders. We aimed to investigate the role of EndMT in abdominal aortic aneurysms (AAA) and the underlying mechanism.

**Methods:** Immunoblotting, immunofluorescence and transmission electron microscope were used to assess d-flow-induced EndMT in human and mouse AAA models (Ang II/PPE). An Ibidi pump system was used to produce d-flow on human aortic endothelial cells (HAECs), and the expression of galectin-7 was enhanced and weakened using an adeno-associated virus. Furthermore, single-cell RNA sequencing was performed to explore the underlying mechanism of galectin-7-mediated EndMT.

**Results:** EndMT induced by d-flow, which suppressed galectin-7 expression, was positively correlated with AAA. Enhanced galectin-7 expression inhibited d-flow-induced EndMT and AAA progression, whereas reduced galectin-7 expression resulted in the opposite effect. Mechanistically, we found a EndMT-related cluster in HAECs by single-cell RNA sequencing, and the SRGN gene in this cluster was considered the core gene. Galectin-7 bound competitively to the transcription factor CREB, resulting in the inhibition of SRGN transcription, which in turn prevented TGFβ/smad pathway activation, thereby restoring EndMT progression.

**Conclusions:** EndMT transformation in ECs exposed to d-flow was the critical driver of AAA development. Furthermore, endothelium-enriched galectin-7 suppressed the EndMT process induced by d-flow and prevent AAA progression by transcriptionally inhibiting SRGN via competitive binding with CREB to restrict TGFβ/smad pathway.

## Introduction

Abdominal aortic aneurysm (AAA) was a progressive and detrimental vascular inflammatory disorder that can result in catastrophic rupture events and sudden death^1^. A variety of risk factors, including genetic mutations^2^, chronic diseases^3^ and environmental change^4^, can lead to the development of AAA, especially hypertension, which is a representative environmental cause of AAA^5^. Although some previous studies had explored that hypertension was highly correlated with the occurrence and development of AAA through its influence on smooth muscle cell phenotype, extracellular matrix degradation and circulating monocyte populations^6^-^8^, the pathological mechanism through which hypertension induces aneurysm through its negative effect on vascular endothelium, the first barrier, is largely unclear. Recent studies have shown that endothelial cell (EC) dysfunction, represented by endothelial-to-mesenchymal transition (EndMT), is caused mainly abnormal shear stress in patients exposed to hypertensive conditions and is an early and critical pathological event of AAA^9^. High blood pressure can produce different shear stress effects on the arterial endothelium at different locations^10^. In the hypertensive state, ECs in the branched and curved areas of the artery are exposed to the disturbed flow (d-flow) and stimulated by oscillatory shear stress (OSS); in contrast, in unbranched and straight arteries, ECs are exposed to laminar flow (L-flow) and stimulated by laminar shear stress (LSS).* In vitro* evidence has revealed that fluid mechanics lead to irregular shapes and sizes of endothelial cells at the top of aneurysms, increasing the risk of rupture^11^. Increasing evidence has revealed that d-flow in the presence of hypertension can trigger EC activation of EndMT, which refers to the loss of endothelial characteristics and acquisition of mesenchymal characteristics, including morphological changes in ECs and decreased cell adhesion function; additionally it has been shown to be a critical driver of vascular inflammation and to further aggravate the development of atherosclerosis^12^-^14^. These findings suggest that exploring the role of d-flow-induced EndMT in aneurysms and the underlying mechanisms may lead to a new therapeutic strategy for AAA.

It is believed that the stress sensitive proteins of vascular endothelium are the first to sense changes in blood flow stress and further lead to EndMT^15^. Galectins are considered to be flow stress-sensing proteins and are widely involved in various vascular endothelium dysfunctions, including adhesion, migration and differentiation disorders^16^, ^17^. Galectin-1, a key regulator of the proteasomal degradation of Ca_V_1.2 channels, plays a unique modulatory role in regulating blood pressure^18^. Similarly, galectin-3 has been found to be the pivotal target gene through which mineralocorticoid receptor activation affects smooth muscle cells^19^. More importantly, galectin-7 plays pivotal roles in tissue repair, preeclampsia and cancer, in which epithelial cells lose differentiation ability while they acquire migratory mesenchymal phenotypes^20^-^22^. Moreover, there is a close interaction between galectin-7 and the TGF-β pathway, which plays a key role in the EndMT process^23^-^25^. Previous studies have indicated that galectin-7 is specifically expressed in human arterial intima^26^. These findings indicated that galectin-7 may play a critical role in the adaptation of large arteries to biomechanical stress and the regulation of EndMT processes in AAA. However, the contribution of galectin-7 to d-flow-induced EndMT and the formation of AAA remains unclear.

Here, we demonstrated that d-flow-induced EndMT occurred in human and mouse AAA tissues. Moreover, the expression of arterial galectin-7 was regulated by different blood flow patterns, with L-flow increasing galectin-7 expression and d-flow decreasing galectin-7 expression both *in vivo* and *in vitro*. Galectin-7 was shown to have the potential to inhibit the EndMT induced by d-flow and prevent AAA progression. Single-cell sequencing and molecular biology experiment revealed that galectin-7 inhibited endothelial cell subsets enriched with SRGN and subsequently blocked the activity of the TGF-β/smad signaling pathway to inhibit the EMT process.

## Materials and Methods

### Ethics statement

The collection and use of human samples were conducted under a protocol approved by the Ethics Committee of Nanfang Hospital, Southern Medical University. All participants provided written informed consent in accordance with the Declaration of Helsinki. The animal experiments were performed strictly in accordance with the recommendations in the Guide for the Care and Use of Laboratory Animals of the National Institutes of Health. All experimental procedures involving animals were reviewed and approved by the Animal Care and Use Committee of Southern Medical University (IACUC-LAC-20231029-001). The mice were anesthetized with an isoflurane/oxygen mixture (2% inhalation). Mice were euthanized by cervical dislocation under anesthesia.

### Human aortic tissue samples

The samples were collected retrospectively from patients who had already undergone AAA or AD surgery, and these resected tissues were stored in a biological database. Immediately after collection, all the samples were frozen in liquid nitrogen and maintained at -80 °C until further processing. Informed consent for this multicenter clinical research project was obtained from the Ethics Committee of Nanfang Hospital. The patient's clinical information is present in Table S1.

### *In vivo* AAV viruses (adeno-associated viruses) injection

The Animal Care and Use Committee of Southern Medical University approved all animal experimental protocols. In addition, in accordance with the National Institutes of Health Laboratory Animal Care and Use Guidelines, we used 10-to 12-week-old male C57BL/6J mice to construct a PPE-induced AAA model and 12-to 16-week-old male ApoE^-/-^ mice to construct an AngII-induced AAA model as described previously^27^. The mice were housed under a 12 h-12 h dark-light cycle at an ambient temperature of 22°C and 60-65% humidity. AAV2-VEC-TIE-ZsGreen, AAV2-VEC-TIE-m-Lgals7-3xflag-ZsGreen, AAV2-VEC-TIE-ZsGreen, AAV2-VEC-TIE-mir30-m-Lgals7-ZsGreen were synthesized by Hanheng Bioscience Technology Co., LTD. (Shanghai, China). ApoE-/ and C57BL/6J mice were injected with AAV2-VEC-TIE-ZsGreen, AAV2-VEC-TIE-m-Lgals7-3xflag-ZsGreen, AAV2-VEC-TIE-ZsGreen, AAV2-VEC-TIE-mir30-m-Lgals7-ZsGreen via the tail vein (1.5×10^12^ viral genome particles per mouse) using an insulin needle. After 30 days, the AAA model was established.

### AAA model induced by Ang II or PPE

On the basis of a previous study^27^, we selected 12- to 16-week-old male ApoE^-/-^ mice for this study. After the mice were anesthetized, an osmotic minipump (Alzet, Model 2004, DURECT Corporation, Cupertino, CA) was implanted subcutaneously in the dorsal neck region, and Ang II (A9525, Sigma) and saline (0.9% NaCl) were administered at a rate of 1 μg/kg/min for 28 days. For the PPE model, 10- to 12-week-old male C57BL/6J mice were completely anesthetized, and then, laparotomy was performed. The abdominal aortic segment between the renal and iliac arteries was isolated, and then, the surface of the abdominal aortic segment was covered with cotton gauze soaked with 40 μL elastase (E1250, Sigma-Aldrich) or saline (0.9% NaCl) for 15 minutes. After 2 weeks, samples were taken for subsequent analyses. To demonstrate the presence of aortic aneurysms, the mice were euthanized. The mice were fixed by systemic perfusion with 4% paraformaldehyde at physiological pressure. The aorta was carefully exposed under a dissecting microscope. The surrounding periadventitial tissue was meticulously cleared, after which the aorta was photographed using a digital camera. The maximal external diameter of the suprarenal aorta (Ang II model) or infrarenal aorta (PPE model) was measured using Image-Pro Plus software (Media Cybernetics). The suprarenal aorta was identified as the passage below the last pair of intercostal arteries and above the right renal branch. An aneurysm was defined as a ≥50% increase in maximal aortic diameter compared with that of control subjects. AAA assessments were conducted by two investigators blinded to the experimental groups.

### Partial ligation of the suprarenal abdominal aorta

Briefly, the mice were anesthetized and fixed to an operating bed, followed by abdominal hair removal and disinfection. A midline abdominal incision was made, and the tissues surrounding the abdominal aorta were dissected. The renal artery and abdominal aorta were isolated and exposed, and a sterile blunt needle was placed parallel to the abdominal aorta superior to the origin of the renal artery, followed by partial ligation of the suprarenal abdominal aorta using absorbable 6.0 silk sutures. The needle was subsequently removed, resulting in standardized partial stenosis. The skin was closed with 5.0 silk sutures, and the animals were observed until recovery after surgery in a chamber on a heating pad.

### Endothelial cell lineage tracing

Lineage tracing of endothelial cells and their progeny was performed using Cdh5-CreERT2(+/-); Rosa26-LSL-tdTomato(+/-) double-transgenic mice supplied by Cyagen Biosciences (Jiangsu, China). Tamoxifen induction activates Cre recombinase exclusively in cells expressing vascular endothelial cadherin (Cdh5), mediating the excision of the floxed STOP cassette (LoxP-Stop-LoxP, LSL) at the Rosa26 locus and resulting in the constitutive expression of the red fluorescent protein tdTomato. Six- to eight-week-old mice received daily intraperitoneal injections of tamoxifen (dissolved in corn oil at 20 mg/mL) at 75 mg/kg body weight for 3 consecutive days. Seven days after the first tamoxifen injection, the mice underwent either sham surgery or PPE modeling. The following primers were used for detection: For tdTomato (+): (F): 5'-GGCATTAAAGCAGCGTATCC-3'; (R): 5'-CTGTTCCTGTACGGCATGG-3'; For tdTomato (-): (F): 5'-AAGGGAGCTGCAGTGGAGTA-3'; (R): 5'-CCGAAAATCTGTGGGAAGTC-3'; For CreERT2: (F): 5'-CACTGGGTCCTGATGGTGCCTATC-3'; (R): 5'-TCCTGTTGTTCAGCTTGCACCAG-3'.

### Aortic aneurysm ultrasonography

After 28 days of Ang Ⅱ infusion, the mice were anesthetized with 2% isoflurane. Abdominal aorta B-mode ultrasound imaging was performed with a Vevo 2100 system (Visual Sonics, ON, Canada).

### Histological analysis

After euthanasia, the whole artery was flushed with saline and subsequently perfusion-fixed with 4% paraformaldehyde. The entire aorta from the aortic arch to the iliac bifurcation was then carefully dissected and excised under a surgical microscope to avoid mechanical stretching or damage. Perivascular adipose and connective tissues were meticulously removed. The aortic segment was further divided into suprarenal and infrarenal regions. The samples were fixed with 4% paraformaldehyde and subsequently embedded in paraffin. Paraffin samples were serially sectioned (5 μm) for elastin van Gieson staining and immunohistochemistry.

#### Elastin staining

Victoria blue van Gieson staining was performed using a commercial kit (GenMed, Qingdao) in accordance with the manufacturer's instructions. Suprarenal or infrarenal aortic samples from the different groups of mice were embedded in paraffin and cut. Paraffin sections (5 μm) were used for elastin staining. The score was based on the previously established elastin degradation criteria: 1, no elastin degradation; 2, mild elastin degradation; 3, moderate elastin degradation; 4, severe elastin degradation with fragmentation or loss or aortic rupture.

### *In vivo* treatment with an EMT inhibitor or an EMT agonist

Mice were assigned randomly to groups and administered a neutralizing antibody targeting TGF-β1, -2, or -3 (BioXCell, BE0057) at a dosage of 250 μg via intraperitoneal injection every other day, or with IgG (BioXCell, BE0083) as a control. To induce EMT, recombinant mouse TGF-β1 protein (R&D Systems, 7666-MB-005) was administered at a dosage of 10 ng via intraperitoneal injection every other day; vehicle injection was used as a control.

### HAECs culture and transfection

Human aortic endothelial cells (HAECs, iCell-0015a) were cultured in in endothelial growth medium (iCell-0015a-001b) in humidified air with 5% CO2 at 37 ℃ in accordance with the manufacturer's instructions. For small interfering RNA (siRNA) or overexpression plasmid transfection, when cells reached 70% to 80% confluence, serum starvation treatment was performed. Prior to transfection, the cells were subjected to serum starvation for 12 hours using basal medium without growth supplements to synchronize the cell cycle and enhance transfection efficiency. The cells were then transfected with an siRNA against lgals7 or a lgals7 overexpression plasmid (oe-lgals7) using Lipofectamine 3000 (Invitrogen, Thermo Fisher Scientific). After incubation for 6 h, the medium was replaced with complete medium, and the cells were cultured for 24 h to allow sufficient gene silencing or overexpression. Then, subsequent experiments were performed.

### *In vitro* shear stress experiments

The shear stress experiments were performed as previously described^14^. Passage 4-6 human aortic endothelial cells (HAECs) were seeded into µ-slide I^0.4^ Luer ibiTreat chambers (Ibidi, Cat. No. 80176), when the cells reached a confluence of 80%, disturbed flow (4 dynes/cm^2^, 1Hz) or lamina flow (12 dynes/cm^2^) was applied to the cells for 24 hours using the Ibidi pump system (IBIDI, Gräfelfing, Germany). Unidirectional constant laminar flow was used to mimic stable blood flow profile in straight vessels. Oscillatory flow was used to mimic disturbed blood flow in arterial bifurcations or curvatures. After the experiment, the cells were processed for subsequent analysis.

### MAEC isolation

MAECs were isolated as described in a previous study^14^, ^28^. The mice were anesthetized, and a midline incision was made to expose the chest and abdomen. The vasculature was perfused by cardiac puncture with 10 ml of PBS containing 20 U/ml heparin, and the entire aorta from the aortic arch to the distal abdominal aorta was carefully dissected under a surgical microscope to minimize mechanical damage. The vessel was cleared of surrounding adipose and connective tissues in ice-cold PBS and divided into three anatomical segments, i.e., the aortic arch, thoracic aorta, and infrarenal abdominal aorta, and each segment was cut into approximately 1 mm^3^ pieces. These pieces were digested in DMEM containing 2 mg/ml collagenase II for 120 minutes at 37 °C under constant shaking. After digestion, the cell suspension was sequentially filtered through 70 μm and 40 μm cell strainers to remove undigested fragments and large debris. The filtered cells were subsequently washed twice with cold PBS supplemented with 2% FBS to inhibit further enzymatic activity and protect cell integrity. Endothelial cells were labeled with antibodies against CD31 and sorted using a cell sorter. To ensure reproducibility and cell purity, the sample handling time was minimized throughout the process, and each sorting session was validated using isotype controls. Only samples whose viability exceeded 95% (assessed by Trypan blue exclusion) and whose sorting purity was >90% were used for subsequent analyses.

### Quantitative real-time PCR

Total RNA was extracted from endothelial cells and aortic tissues using TRIzol reagent (Invitrogen, 15596026) in accordance with the manufacturer's protocol. cDNA was reverse transcribed from total RNA using PrimeScript^TM^ RT Master Mix (TaKaRa Biotechnology, Dalian, China). For reverse transcription, strand-specific and reverse primers were used. Quantitative real-time PCR (qRT-PCR) was performed with a SYBR Green RT-PCR Kit (Takara Biotechnology) using Light Cycler 480 II equipment (Roche Diagnostics, Basel, Switzerland). The 2-ΔΔCt method was used to normalize the expression of related genes to that of glyceraldehyde-3-phosphate dehydrogenase. (GAPDH) mRNA expression was used as an internal control. The primer sequences are presented in Table S2.

### Western blotting

The western blots procedure was performed as previously described^29^. Protein from endothelial cells and aortic tissues was extracted in ice-cold radioimmunoprecipitation assay (RIPA) lysis buffer (Beyotime, P0013B), supplemented with protease (Beyotime, ST506) and phosphatase (Beyotime, P1081) inhibitors to maintain protein integrity and phosphorylation status. The extracted proteins were electrophoresed and then transferred to a polyvinylidene fluoride (PVDF) membranes. Protein molecular weight markers (Thermo Fisher Scientific, 26616 and 26625) were used for reference. Following transfer, the membranes were blocked against nonspecific binding by incubation in 5% BSA for 1 h at 37 °C. They were subsequently probed with primary antibodies at 4 °C overnight. After three 10-minute washes with TBST, the membranes were incubated with secondary antibodies for 1 hour at room temperature.

The protein bands were visualized by an enhanced chemiluminescence (Advance, No. RPN2235, GE Healthcare Life Sciences) and intensities were quantified using ImageJ software, with β-actin serving as the loading control. The antibodies used are listed in Table S7.

### Chromatin Immunoprecipitation (ChIP)

HAECs grown in vitro were collected and subsequently employed for ChIP experiments. ChIP were performed using EZ-ChIP Chromatin Immunoprecipitation Kit (Milipore) according to the manufacturer's protocol. In brief, HAECs were first fixed and then lysed, and sonication was performed until the desired length was reached. Then, an anti-CREB antibody (5 μg), or a control IgG antibody (5 μg) was used for ChIP. Next, DNA fragments were quantified by gel electrophoresis. To quantify CREB binding to the SRGN promoter, the DNA samples after ChIP were analyzed by q-PCR with specific primers (Table S2). The antibodies used are presented in Table S6.

### Immunohistochemistry analysis

Paraffin sections were deparaffinized, after which antigen retrieval was performed. Endogenous peroxidase activity was blocked with 3% hydrogen peroxide, and nonspecific binding sites were blocked by incubating the sections with 10% bovine serum. Then, the sections were incubated with primary antibodies overnight at 4 °C. The following day, the sections were washed three times with PBS and incubated with a biotin-conjugated secondary antibody for 1 h at room temperature, followed by incubation with horseradish peroxidase-labeled streptavidin solution. The sections were stained with diaminebenzidine and counterstained with hematoxylin. The antibodies used are listed in Table S3.

### Immunofluorescent staining

Tissues or cultured cells were fixed with 4% paraformaldehyde for 30 min at room temperature. After fixation, the samples were washed three times (5 minutes each) with PBS. Then, the samples were permeabilized with 1% Triton X-100 for 10 min and blocked with 5% BSA for 1 h. After blocking, the samples were incubated overnight at 4 °C with primary antibodies. The following day, the samples were washed three times with PBS (5 minutes each) and incubated with species-appropriate fluorescently conjugated secondary antibodies for 1 h at room temperature in the dark. Following incubation with a secondary antibody, the samples were washed three times with PBS (5 minutes each). Nuclei were counterstained with DAPI for 10 min at room temperature, followed by two additional washes with PBS. All the samples were stored at 4 °C in the dark until imaging. The antibodies used are listed in Table S4.

### Co-immunoprecipitation (Co-IP) assays

In the Co-Immunoprecipitation (Co-IP) procedure, cells were first lysed using IP lysis buffer. The primary antibody was then conjugated to the protein samples and incubated at 4 °C for an extended period, typically overnight. Then, Protein A/G beads were added to each sample, and the lysate and beads mixture was incubated at 4 °C with continuous rotation for approximately 3 h to ensure thorough agitation. After the immunoprecipitation process, the samples underwent three rounds of washing with IP lysis buffer to remove any unbound proteins. Finally, the proteins of interest were eluted using 1x SDS sample buffer, followed by SDS-PAGE, Western blotting. The antibodies used are listed in Table S5.

### Single-cell RNA sequencing

After the samples were processed, single-cell isolation and library preparation were performed. Next, we performed sequencing, followed by data analysis. In the analysis of single-cell RNA sequencing data, a suite of quality filters was implemented to exclude cells that met any of the following criteria: abnormally low number of detected genes, abnormally high number of genes or UMIs, and a high mitochondrial gene percentage. Using the filtered cell gene expression matrix, Cell Ranger was employed to perform cell typing by dimensionality reduction and clustering. PCA was performed first, followed by clustering. Cell Ranger was used in conjunction with the negative binomial test to identify differentially expressed genes between distinct cellular clusters. Functional enrichment analysis was conducted by GO functional enrichment and KEGG pathway enrichment analyses. Significant enrichment was considered a p_adj_ value less than 0.05.

### Statistical analysis

Our results are presented as the mean ± SD. The elastin degradation scores are expressed as medians and quartiles. For continuous variables, normality was assessed using the Shapiro-Wilk test. For data with homogeneous variance across the different groups, Student's t-test was employed to assess significant differences between two groups. For comparisons among multiple groups, one-way ANOVA followed by a post hoc Bonferroni's multiple comparisons test for multiple comparisons was used. If the variables were not normally distributed, the Mann-Whitney test was used for two groups, and the nonparametric Kruskal‒Wallis test with Dunn's multiple comparison test for multiple independent groups was used. Comparison of AAA incidence were performed using Fisher's exact test, and the log-rank (Mantel-Cox) test was used for survival analysis. The data were analyzed with GraphPad Prism 8. A p-value < 0.05 was considered to indicate statistical significance.

## Results

### Disturbed flow-induced EndMT was detected in human and mouse AAA tissues

**Figure 1:**
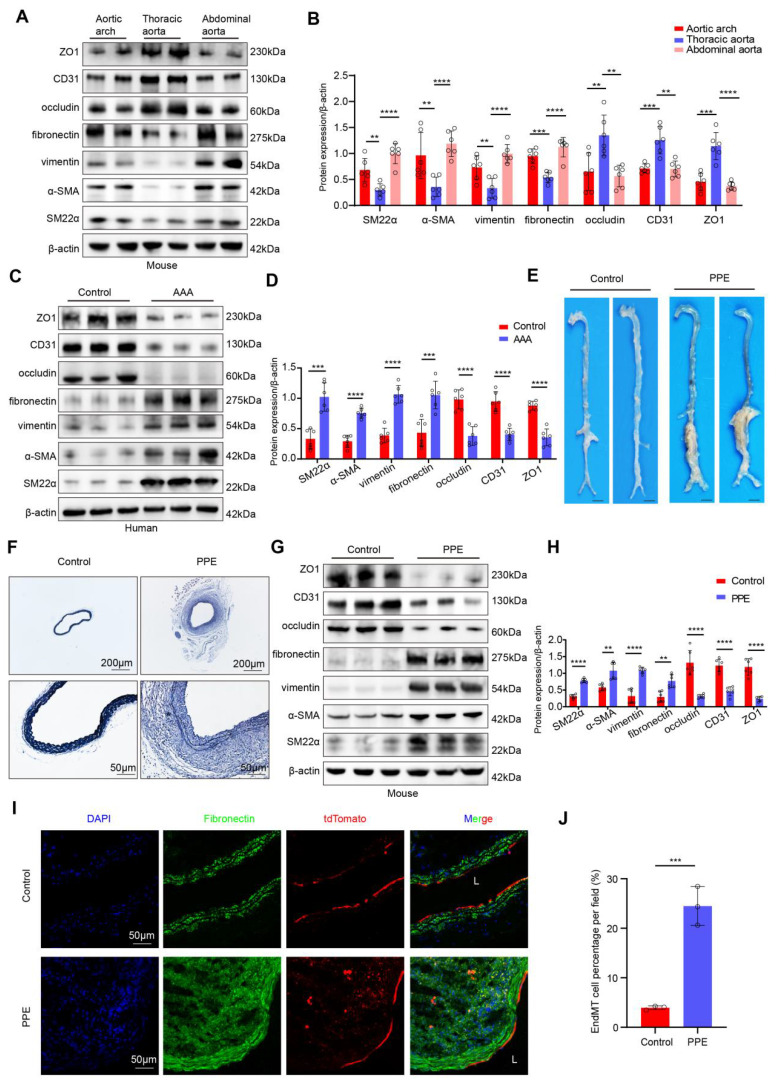
** D-Flow-induced EndMT is present in human AAA samples and a mouse model of PPE-induced AAA. A**-**B**, Western blotting and densitometric analysis of SM22α, α-SMA, vimentin, fibronectin, occludin, CD31 and ZO1 protein expression levels in the endothelium of the aortic arch, thoracic and abdominal aorta (n = 6/group). **C**-**D**, Western blotting and densitometric analysis of SM22α, α-SMA, vimentin, fibronectin, occludin, CD31 and ZO1 protein expression levels in the endothelium of human AAA samples and adjacent control aortas (n = 6/group). **E**, Representative macroscopic photographs of the infrarenal abdominal aorta from control and PPE-induced AAA mice. **F**, Representative EVG staining images of the mouse aortas (scale bars = 200μm and 50μm, n = 8/group). **G**-**H**, Western blotting and densitometric analysis of SM22α, α-SMA, vimentin, fibronectin, occludin, CD31 and ZO1 protein expression levels in the endothelium of control aortas and PPE**-**induced AAA (n = 6/group). **I-J**. Representative immunofluorescence images and corresponding densitometric analysis of abdominal aortas from Cdh5-CreERT2 mice (scale bars = 50 μm, n = 3/group). One-way ANOVA with a post hoc Bonferroni's multiple comparisons test was performed for B. Unpaired Student's t-test was performed for D, H and J. *p < 0.05, **p < 0.01, ***p < 0.001, and ****p < 0.0001.

To explore whether the occurrence of EndMT is associated with different hemodynamic patterns, we extracted proteins from endothelial cells of the aortic arch, thoracic aorta, and abdominal aorta in mice and compared the expression levels of endothelial and mesenchymal markers. Compared with those in the L-flow-exposed thoracic aorta endothelial cells, the expression of occludin, CD31 and ZO1 was lower and the expression of SM22α, α-SMA, vimentin and fibronectin was higher in d-flow-exposed aortic arch and abdominal aorta endothelial cells (**Figure 1A-B**). *In vitro* western blot results indicated that compared with static controls and LSS-exposed HAECs, OSS-exposed HAECs exhibited significantly higher expression of mesenchymal markers and significantly lower expression of endothelial markers. However, compared with static control HAECs, LSS-exposed HAECs did not significantly differ (**Figure S1A-B**). These results suggested that EndMT was induced by disturbed flow. Furthermore, we obtained human AAA tissues to determine whether disturbed flow-induced EndMT occurs in AAA. Western Blotting revealed that the expression levels of occludin, CD31 and ZO1 were lower and the expression levels of SM22α, α-SMA, vimentin and fibronectin were higher in endothelial cells from human AAA samples than in corresponding adjacent normal aortic tissues (**Figure 1C-D**). Consistent with the results for human AAA samples, both the elastase-induced infrarenal and the AngII-induced suprarenal mouse AAA models had lower expression of endothelial markers and higher expression of mesenchymal markers than their respective controls did (**Figure 1E-H, Figure S2A-E**). Immunofluorescence staining revealed that the proportion of ECs expressing vimentin or fibronectin was significantly greater in the vascular endothelium of both AAA models than in that of their respective controls (**Figure S2F-I, Figure S3A-D**). In addition, transmission electron microscopy revealed the occurrence of EndMT in Ang II-induced AAA, including impaired tight junctions in ECs and morphological changes in ECs (**Figure S2J-K**). To confirm the presence of endothelial-mesenchymal transition (EndMT), we generated endothelial lineage-tracing mice (**Figure S4A-C**) and established a PPE-induced AAA model. The results revealed that the tdTomato fluorescence in the sham group was confined to the endothelial layer, whereas in the PPE group, the red fluorescence of tdTomato was observed in the arterial interstitium, suggesting that these cells originated from endothelial cells that underwent EndMT (**Figure 1I-J**). These results indicated that disturbed flow-induced EndMT occurs in AAA and may be involved in AAA progression.

### AAA progression was significantly increased after EndMT induced by disturbed flow

**Figure 2:**
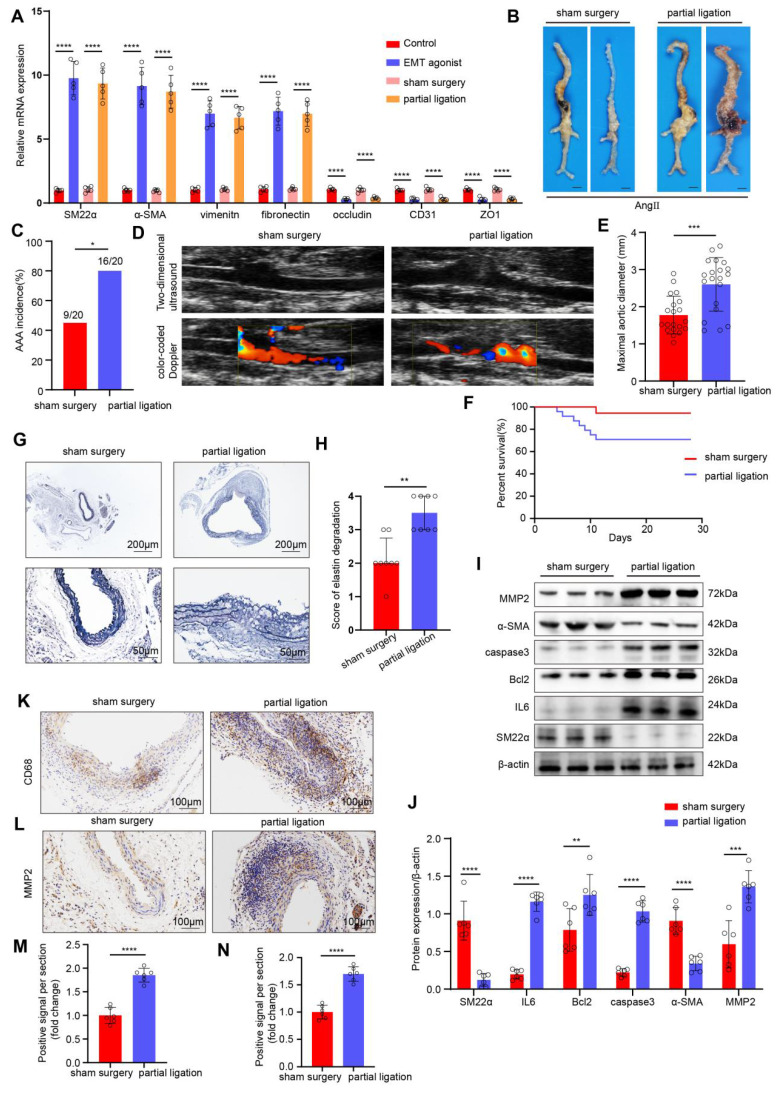
** AAA progression significantly increases after EndMT is induced by disturbed flow. A,** The relative PCR expression of SM22α, α-SMA, vimentin, fibronectin, occludin, CD31 and ZO1 in the 4 indicated groups (n = 5/group). **B**, Representative macroscopic photographs of suprarenal AAA induced by AngII at 28 d. **C**, AngII-induced AAA incidence in the two indicated groups (n = 20/group). **D**, Two-dimensional ultrasound and color-coded Doppler imaging of aortas after sham surgery and partial ligation in AngII-induced AAA model mice. **E**, The maximal abdominal aortic diameter in the two indicated groups (n = 20/group). **F**, Survival curves of AngII-infused ApoE^-/-^ mice in the two indicated groups (n = 20/group). **G-H**, Representative images of EVG staining and elastin degradation scores in the two indicated groups (scale bars = 200μm and 50μm, n = 8/group). **I-J**, Western blot and densitometric analyses of SM22α, IL6, Bcl2, caspase3, α-SMA and MMP2 protein expression in the two indicated groups (n = 6/group). **K-N**, Immunohistochemical staining and corresponding densitometric analysis of CD68 (K) and MMP2 (L) in the two indicated groups (scale bars = 100μm, n = 6/group). One-way ANOVA with a post hoc Bonferroni's multiple comparisons test was performed for A. Fisher's exact test was performed for C. Unpaired Student's t-test was performed for E, J and M-N. Mann-Whitney test was performed for H. *p < 0.05, **p < 0.01, ***p < 0.001, ****p < 0.0001.

Next, we investigated the effects of disturbed flow-induced EndMT on the formation of AAA. We constructed an *in vivo* disturbed flow model by partial ligation of the suprarenal abdominal aorta in ApoE^-/-^ male mice. To demonstrate the successful induction of EndMT by in vivo disturbed flow, we divided the mice into the following groups: control group, EMT agonist group, the sham surgery group, and the partial ligation of the suprarenal abdominal aorta group. As determined by PCR, compared with the sham surgery group, in the partial ligation group, the expression of mesenchymal markers in the endothelium of the suprarenal and infrarenal abdominal aorta were significantly higher, and the expression of endothelial markers was significantly lower, indicating that the *in vivo* disturbed flow model successfully induced EndMT (**Figure 2A**). Next, an AngII-induced mouse AAA model was established, and the mice were divided into a sham surgery group and a partial ligation of the suprarenal abdominal aorta group. The incidence of Ang II-induced AAA was 45% in the sham surgery group versus 80% in the ligation group (**Figure 2B-C**). On the 28th day of AngII infusion, vascular ultrasound imaging revealed more obvious dilation of the abdominal aorta in the mice in the ligation group than in the mice in the sham surgery group (**Figure 2D**). The maximal diameter of abdominal aorta significantly increased (**Figure 2E**), and the cumulative survival markedly decreased in the ligation group (**Figure 2F**). EVG staining revealed that the elastic fibers in the ligation group were disrupted (**Figure 2G-H**). Western blot results indicated that ligation increased IL6, Bcl2, caspase3 and MMP2 levels and decreased the SM22α and α-SMA levels (**Figure 2I-J**). Additionally, the immunohistochemical results revealed that the expression levels of CD68 and MMP2 in the ligation group were greater than those in the sham surgery group (**Figure 2K-N**). To directly confirm the effect of disturbed flow (d-flow) created by ligation from the systemic effects of Ang II, a control group comprised mice that underwent partial ligation but were administered saline were added. In these mice, we did not observe significant AAA formation despite the presence of any potential compensatory RAS effects (**Figure S5A-D**). Together, these results demonstrated that AAA progression significantly increased after EndMT induced by disturbed flow.

### Flow-related endothelial galectin-7 expression was downregulated during AAA

**Figure 3:**
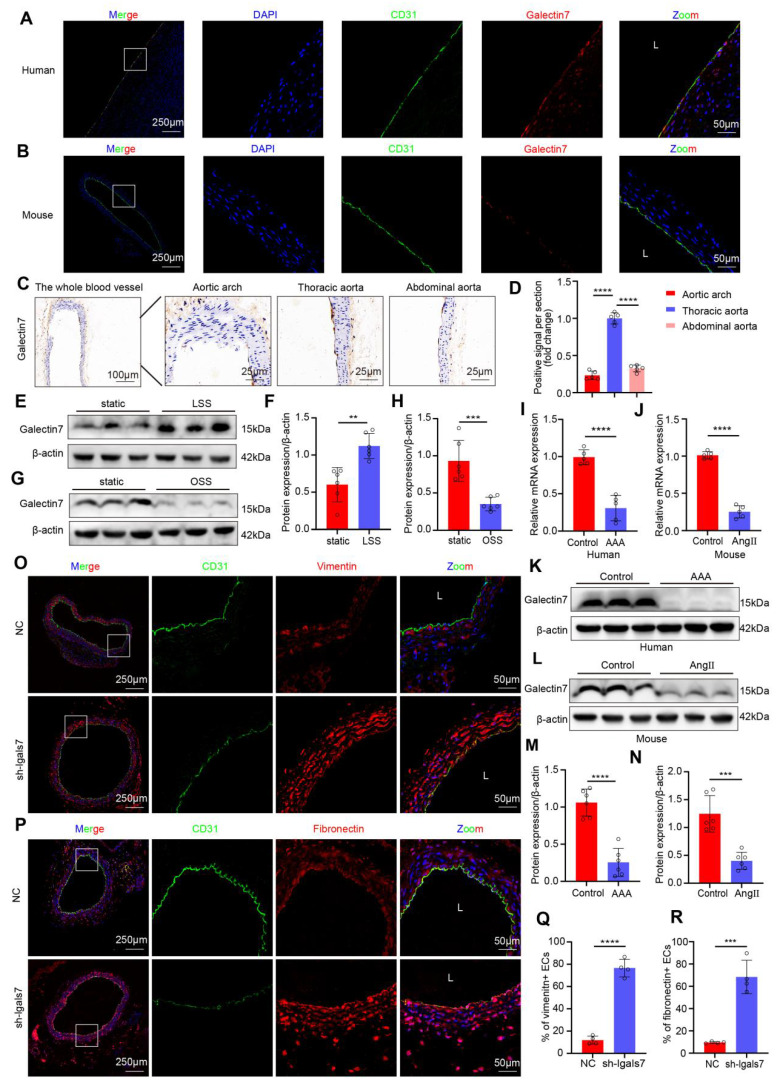
** Galectin-7 is a flow-sensitive protein, and galectin-7 knockdown promotes EndMT *in vivo*. A-B**, Double immunofluorescence staining for CD31 (green) and galectin-7 (red) of human aorta (A) and mouse aorta (B). Nuclei were co-stained with DAPI (blue) (scale bars = 250μm and 50μm, n = 4/group). **C-D**, Representative images and densitometric analysis of immunohistochemical staining of galectin-7 in the aorta. Galectin-7 expression was lower in the aortic arch and abdominal aorta than in the thoracic aorta (scale bars = 100μm and 25μm, n = 5/group). **E-F**, Western blot and densitometric analyses of galectin-7 protein expression levels in HAECs under LSS and static conditions (n = 6/group). **G-H**, Western blot and densitometric analyses of galectin-7 protein expression in HAECs under OSS and static conditions (n = 6/group). **I-J**, The relative PCR expression of galectin-7 in human AAA samples (I) and mouse AAA samples (J) (n = 5/group). **K-N**, Western blot and densitometric analyses of galectin-7 protein expression in human AAA samples (K, M) and mouse AAA samples (L, N) (n = 6/group). **O-R**, Double immunofluorescence staining for CD31 (green) and vimentin (red) (O) or fibronectin (red) (P) in the sh-NC group and sh-lgals7 group. Nuclei were co-stained with DAPI (blue) (scale bars = 250μm and 50μm, n = 4/group). One-way ANOVA with a post hoc Bonferroni's multiple comparisons test was performed for D. Unpaired Student's t-test was performed for F, H, I-J, M-N and Q-R. *p < 0.05, **p < 0.01, ***p < 0.001, ****p < 0.0001.

Previous studies have demonstrated that the galectin family plays important roles in atherosclerosis and AAA, among which galectin-7 is involved in the tissue re-epithelialization process and might play a significant role in the mechanism through which large arteries accommodate biomechanical stress. Accordingly, we confirmed that galectin-7 was localized in the vascular intima of human aortic samples and mouse aortas by immunofluorescence staining (**Figure 3A-B**). To explore whether different hemodynamic patterns play important roles in modulating the expression level of galectin-7, we isolated the entire blood vessel from the aortic arch of mice for immunohistochemistry. Immunohistochemical results revealed that intimal galectin-7 expression was higher in the thoracic aorta exposed to laminar flow, but lower in the aortic arch and abdominal aorta exposed to disturbed flow (**Figure 3C-D**). Furthermore, we applied different flow treatments to HAECs via the Ibidi pump system *in vitro* to verify the effect of hemodynamics on galectin-7 expression. Galectin-7 protein expression was significantly higher in HAECs exposed to laminar flow (laminar shear stress, 12 dynes/cm^2^) for 24 h than in those exposed to static conditions (**Figure 3E-F**). However, galectin-7 protein expression was significantly downregulated after 24 h of exposure to disturbed flow (oscillatory shear stress, 4 dynes/cm^2^, 1 Hz) (**Figure 3G-H**). These results indicate that galectin-7 is a flow-sensitive protein. In addition, we found that the expression of galectin-7 was lower in human AAA samples and mouse AAA samples than in controls, as determined by qPCR and western blot analyses (**Figure 3I-N**). Interestingly, the qPCR results demonstrated that as the diameter of human AAA specimens increased, the expression levels of galectin-7 generally tended to decrease (**Figure S6**). Next, we used AAV2/VEC-TIE-mir30-m-lgals7 to construct mice with vascular endothelial cell specific galectin-7 knockout. After specific knockout of galectin-7 in ECs, the number of ECs expressing vimentin or fibronectin in the laminar flow-exposed thoracic aorta was significantly greater than that in the control aorta (**Figure 3O-R**). These results indicated that flow-related galectin-7 expression was downregulated during AAA and that the vascular endothelial knockdown of galectin-7 resulted in enhanced EndMT.

### Endothelial galectin-7 prevents AAA formation, and EC-specific galectin-7 knockdown promoted AAA formation

**Figure 4:**
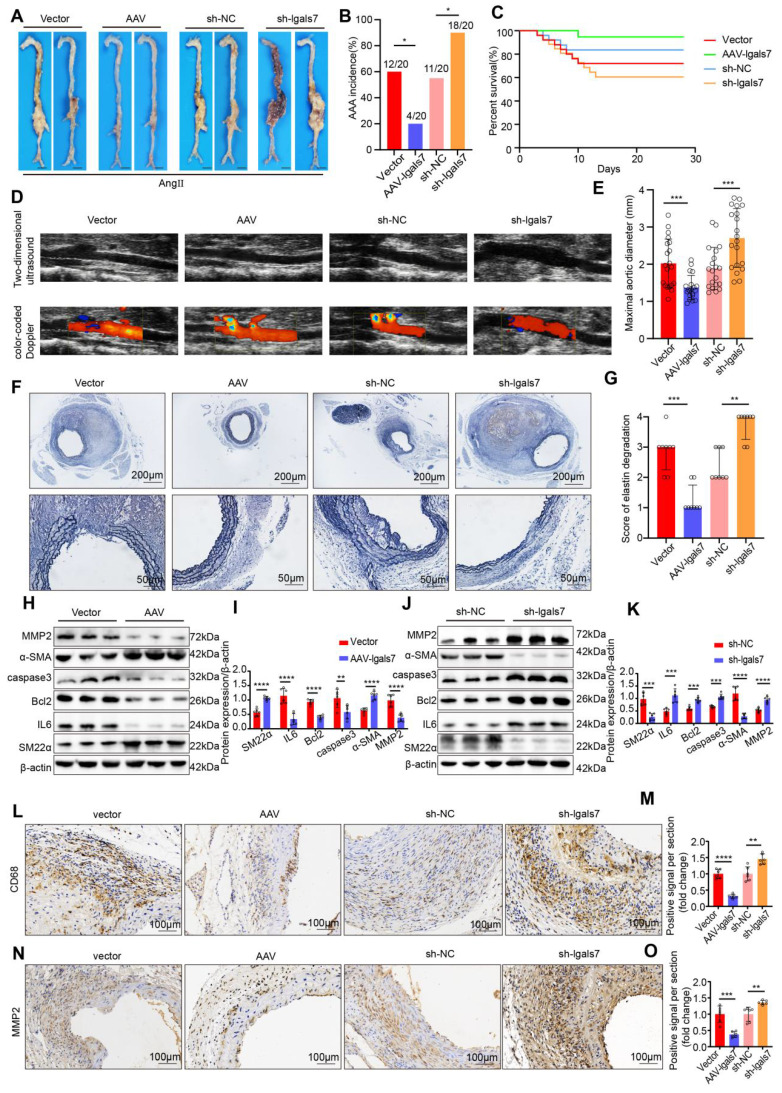
** EC-specific galectin-7 overexpression prevents Ang II-induced AAA formation, and EC-specific of galectin-7 knockdown promotes Ang II-induced AAA formation. A**, Representative macroscopic images of suprarenal AAA in the 4 indicated groups after 28 days of Ang II induction. **B**, Ang II-induced AAA incidence in the 4 indicated groups (n = 20/group). **C**, Survival curves of Ang II-infused ApoE^-/-^ mice in the 4 indicated groups (n = 20/group). **D**, Two-dimensional ultrasound and color-coded Doppler imaging of Ang II-induced AAA in the 4 indicated groups. **E**, The maximal abdominal aortic diameter of Ang II-induced AAA in the 4 indicated groups (n = 20/group). **F-G**, Representative images of EVG staining and elastin degradation scores for the 4 indicated groups (scale bars = 200μm and 50μm, n = 8/group). **H-I**, Western blot and densitometric analyses of SM22α, IL6, Bcl2, caspase3, α-SMA and MMP2 protein expression in Ang II-induced AAA samples in the two indicated groups (n = 6/group). **J-K**, Western blot and densitometric analyses of SM22α, IL6, Bcl2, caspase3, α-SMA and MMP2 protein expression in Ang II-induced AAA samples in the two indicated groups (n = 6/group). **L-O**, Immunohistochemical staining and corresponding densitometric analysis of CD68 (L) and MMP2 (N) in Ang II-induced AAA samples in the 4 indicated groups (scale bars = 100μm, n = 6/group). Fisher's exact test was performed for B. One-way ANOVA with a post hoc Bonferroni's multiple comparisons test was performed for E, M and O. Kruskall-Wallis with Dunn's multiple comparisons test were performed for G. Unpaired Student's t-tests were performed for I and K. *p < 0.05, **p < 0.01, ***p < 0.001, ****p < 0.0001.

To verify the effect of galectin-7 against AAA development, AAV2/VEC-TIE-m-lgals7 and AAV2/VEC-TIE-mir30-m-lgals7 were used to construct vascular endothelial cell-specific overexpression and knockdown of galectin-7, respectively, in male ApoE^-/-^ mice. The incidence of AAA induced by Ang II infusion was 60%, 20%, 55%, and 90% in the vector group mice, AAV-lgals7 group, sh-NC group mice and sh-lgals7 group, respectively (**Figure 4A-B**). Moreover, compared with vector treatment, galectin-7 overexpression significantly improved cumulative survival, whereas compared with sh-NC treatment, galectin-7 knockdown markedly decreased cumulative survival (**Figure 4C**). After 28 days of Ang II infusion, ultrasound revealed slight dilation of the abdominal aorta in the AAV-lgals7 group, whereas the abdominal aorta in the sh-lgals7 group was significantly more dilated than that in the respective controls. (**Figure 4D**). The maximum outer diameter of the abdominal aorta in the AAV-lgals7 group was significantly smaller than that in the vector group, whereas in the sh-lgals7 group, it was significantly larger than that in the sh-NC group (**Figure 4E**). EVG staining revealed that compared with the control group, the AAV-lgals7 group exhibited mild elastic fibers degradation, and compared with the control group, the sh-lgals7 group exhibited elastic fibers disruption (**Figure 4F-G**). The western blot results indicated that galectin-7 overexpression decreased IL6, Bcl2, caspase3 and MMP2 levels and increased the SM22α and α-SMA levels in mouse aortas (**Figure 4H-I**). Conversely, galectin-7 knockdown increased the protein levels of IL6, Bcl2, caspase3 and MMP2 and decreased the protein levels of SM22α and α-SMA in mouse aortas (**Figure 4J-K**). Immunohistochemical results indicated that galectin-7 overexpression reduced CD68 and MMP2 expression in Ang II-induced AAA mice, whereas galectin-7 knockdown increased their expression (**Figure 4L-O**). Furthermore, we performed validation studies after the aforementioned functional experiments using an elastase-induced mouse model of AAA to validate the role of galectin-7 in the progression of AAA, and the results were consistent with those from the AngII-induced AAA model (**Figure S7, 8**). These results indicate that targeting galectin-7 influenced the development of AAA.

### Endothelial galectin-7 inhibited disturbed flow-induced EndMT

**Figure 5:**
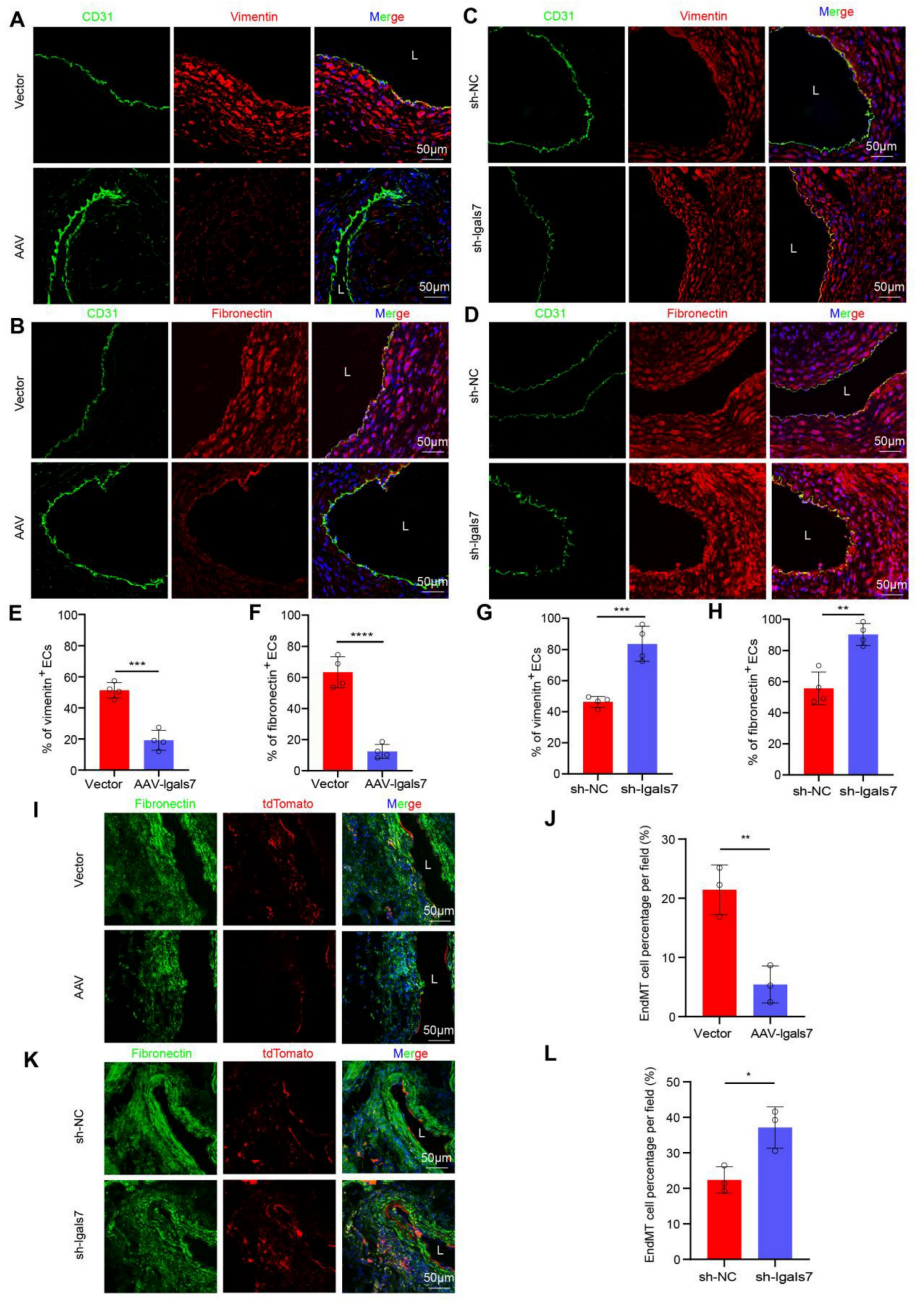
** EC-specific galectin-7 overexpression inhibits disturbed flow-induced EndMT, and EC-specific galectin-7 knockdown promotes disturbed flow-induced EndMT in AngII- and PPE-induced AAA. A** and **E**, Immunofluorescence staining and corresponding densitometric analysis of CD31 (green) and vimentin (red) in the 2 indicated groups. Nuclei were costained with DAPI (blue) (scale bars = 50 μm; n = 4/group). **B** and **F**, Immunofluorescence staining and corresponding densitometric analysis of CD31 (green) and fibronectin (red) in the 2 indicated groups. Nuclei were costained with DAPI (blue) (scale bars = 50 μm; n = 4/group). **C** and **G**, Immunofluorescence staining and corresponding densitometric analysis of CD31 (green) and vimentin (red) in the 2 indicated groups. Nuclei were costained with DAPI (blue) (scale bars = 50 μm; n = 4/group). **D** and **H**, Immunofluorescence staining and corresponding densitometric analysis of CD31 (green) and fibronectin (red) in the 2 indicated groups. Nuclei were costained with DAPI (blue) (scale bars = 50 μm; n = 4/group). **I-J**. Immunofluorescence staining and corresponding densitometric analysis of tdTomato (red) and fibronectin (green) in Cdh5-CreERT2 mouse aortas in the 2 indicated groups (scale bars = 50 μm, n = 3/group). **K-L**. Immunofluorescence staining and corresponding densitometric analysis of tdTomato (red) and fibronectin (green) in Cdh5-CreERT2 mouse aortas in the 2 indicated groups (scale bars = 50 μm, n = 3/group). Unpaired Student's t tests were performed for E-H, J and L. *p < 0.05, **p < 0.01, ***p < 0.001, and ****p < 0.0001.

We next evaluated the role of galectin-7 in disturbed flow-induced EndMT during AAA progression. Compared with their respective control mice, in both AngII-induced suprarenal and elastase-induced infrarenal AAA model mice with endothelial galectin-7 overexpression, immunofluorescence staining revealed significantly fewer ECs expressing vimentin or fibronectin (**Figure 5A-B, E-F; Figure S10A-D**); in contrast, mice in the sh-lgals7 group exhibited a significant increase in the number of ECs expressing these markers (**Figure 5C-D, G-H; Figure S10A-D**). Furthermore, Western blotting revealed that the AAV-lgals7 group had upregulated occludin, CD31, and ZO1 expression but downregulated SM22α, α-SMA, vimentin, and fibronectin expression in both AAA models. Conversely, the sh-lgals7 group exhibited the opposite changes for all these proteins (**Figure S9A-B, Figure S10E-F**). Lineage tracing revealed that compared with that in the arterial medial layer in the control group, the tdTomato red fluorescence signal in the arterial medial layer was reduced in the galectin-7 overexpression group, indicating a decrease in EndMT. In contrast, compared with the control group, the sh-lgals7 group presented a significant increase in the number of EndMT-derived cells within the interstitium (**Figure 5I-L**).

Having established that galectin-7 is a flow-sensitive protein whose expression is decreased by disturbed flow, we next tested its significance for the EndMT of HAECs under disturbed flow conditions by applying OSS to HAECs for 24 h *in vitro* using an Ibidi pump system. Immunofluorescence staining revealed that the proportion of OSS-exposed HAECs expressing SM22α, vimentin, or fibronectin decreased after galectin-7 overexpression. However, this proportion significantly increased after galectin-7 knockdown (**Figure S11A-F**). Western blotting revealed that the expression of occludin, CD31 and ZO1 increased whereas the expression of SM22α, α-SMA, vimentin, and fibronectin decreased in OSS-exposed HAECs with galectin-7 overexpression (**Figure S11G-H**). The opposite trend was observed in OSS-exposed HAECs with galectin-7 knockdown. These results indicate that galectin-7 plays a regulatory role in disturbed flow-induced EndMT.

### Endothelial galectin-7 suppresses AAA progression by inhibiting disturbed flow-induced EndMT

**Figure 6:**
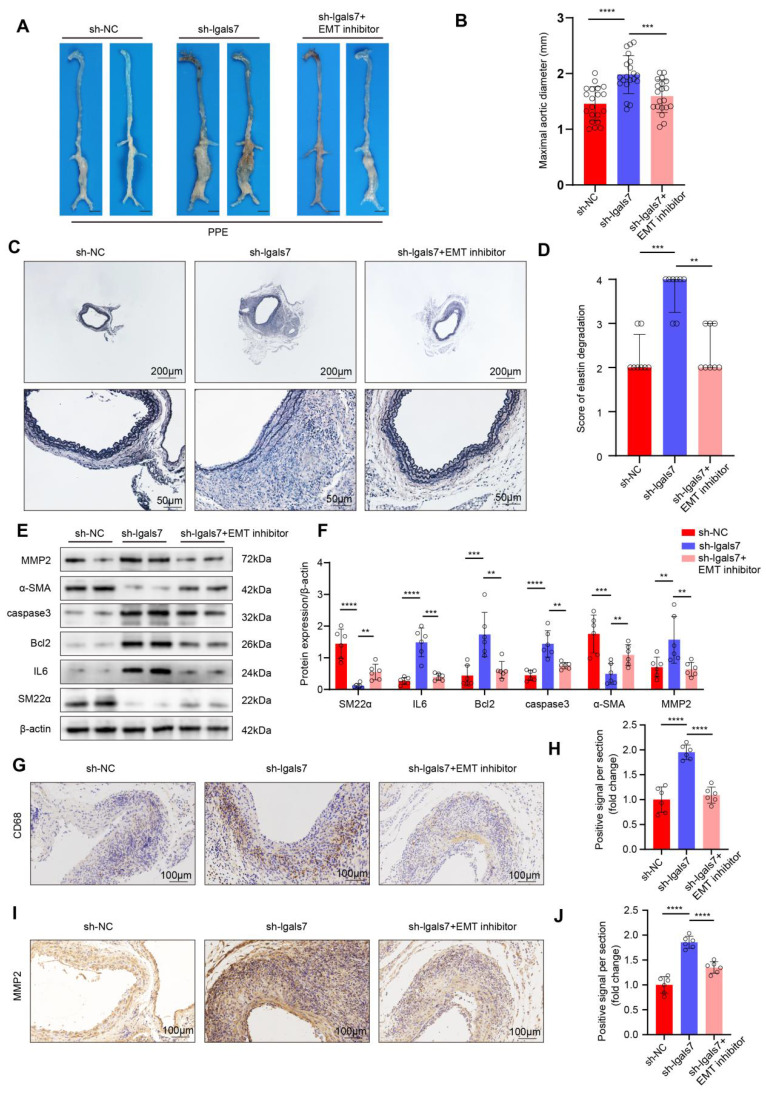
** Endothelial galectin-7 suppresses PPE-Induced AAA progression by inhibiting d-flow-induced EndMT. A**, Representative macroscopy images of infrarenal AAA in the 3 indicated groups after PPE induction. **B**, The maximal abdominal aortic diameter of PPE-induced AAA in the 3 indicated groups (n = 20/group). **C-D**, Representative elastin staining and elastin degradation scores in the 3 indicated groups (scale bars = 200 μm and 50 μm, n = 8/group). **E-F**, Western blot and densitometric analyses of aortic SM22α, IL6, Bcl2, caspase3, α-SMA and MMP2 protein expression in PPE-induced AAA samples (n = 6/group). **G-J**, Representative immunohistochemical staining and corresponding densitometric analysis of CD68 (G) and MMP2 (I) (scale bars = 100 μm; n = 6/group). One-way ANOVA with a post hoc Bonferroni multiple comparisons test was performed for B, F, H and J. Kruskal-Wallis with Dunn's multiple comparisons test was performed for D. *p < 0.05, **p < 0.01, ***p < 0.001, and ****p < 0.0001.

Next, we investigated whether galectin-7 influences AAA formation by regulating disturbed flow-induced EndMT. On the first day following AngII infusion, mice were treated with a control or an EMT inhibitor for 28 days. Western blot results confirmed the efficacy of the EMT inhibitor (**Figure S12A-B**). After 28 days of AngII infusion, the incidence of AAA in mice in the sh-NC group and sh-lgals7 group treated with the control was 50% and 85%, respectively, while the incidence of AAA in mice in the sh-lgals7 group treated with the EMT inhibitor was 50% (**Figure S12C-D**). Moreover, compared with sh-lgals7 treatment, EMT inhibition significantly improved cumulative survival (**Figure S12E**). Vascular ultrasound imaging revealed milder vascular dilation in the EMT inhibitor-treated group than in the sh-lgals7 group (**Figure S12F**). Compared with that in the sh-lgals7 group, the maximal diameter of the abdominal aorta was significantly smaller in the EMT inhibitor group in both the AngII-induced suprarenal and elastase-induced infrarenal AAA models (**Figure 6A-B, Figure S12G**). EVG staining revealed severe elastic fiber disruption in the sh-lgals7 group, an effect that was attenuated by treatment with the EMT inhibitor in both AAA models (**Figure 6C-D, Figure S12H-I**). Western blotting revealed that the expression of IL6, Bcl2, caspase3, and MMP2 was significantly increased whereas the expression of SM22α and α-SMA was decreased in the sh-lgals7 group; these changes were reversed by treatment with the EMT inhibitor in both AAA models (**Figure 6E-F, Figure S12J-K**). Immunohistochemical analysis revealed that galectin-7 knockdown increased CD68 and MMP2 expression in both AAA models and that these effects were abolished by treatment with the EMT inhibitor (**Figure 6G-J, Figure S12L-O**). These results indicated that galectin-7 suppressed AAA progression by inhibiting disturbed flow-induced EndMT.

### Endothelial galectin-7 inhibited disturbed flow-induced EndMT by regulating EndMT-related clusters

**Figure 7:**
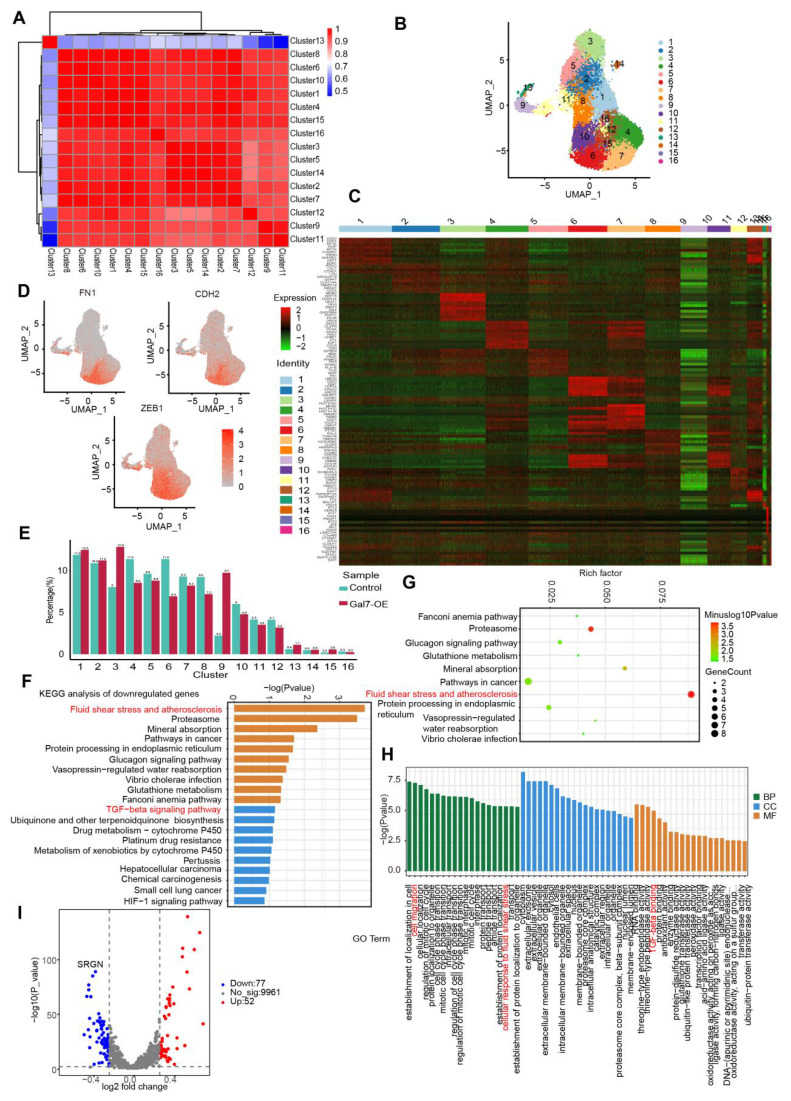
** Endothelial galectin-7 inhibits d-flow-induced EndMT by regulating EndMT-related cluster in HAECs. A**, Principal component analysis (PCA) of single-cell RNA sequencing data. **B**, Uniform manifold approximation and projection (UMAP) plot showing all HAECs colored on the basis of the identified 16 clusters. **C**, Heatmap of unsupervised cluster analysis, characterized by the top 10 differential genes of each cluster. **D**, UMAP feature plot displaying the differential expression of selected EndMT genes. **E**, A bar plot representing the relative proportion of clusters in each sample. **F-G**, KEGG enrichment analysis of downregulated pathways in cluster 6. **H**, Gene ontology (GO) analysis in cluster 6. **I**, Volcano plot exhibiting differentially expressed genes (DEGs) in control HAECs and HAECs overexpressing galectin-7.

To elucidate the mechanism by which galectin-7 inhibits disturbed flow-induced EndMT, we performed single-cell RNA sequencing (scRNA-seq) of OSS-exposed HAECs with or without galectin-7 overexpression. We performed dimensionality reduction on the scRNA-seq data using principal component analysis (PCA) to reveal the heterogeneity among cells and identify distinct cellular groups (**Figure 7A**). Using the PCA results, we utilized UMAP for the visualization of cells and performed clustering analysis, which segregated the cells into 16 distinct clusters (**Figure 7B**). To describe and characterize the clusters of HAECs, we visualized the expression of marker genes specific for each cluster in a heatmap (**Figure 7C**). Furthermore, using mesenchymal biomarkers (FN1, CDH2, ZEB1), we confirmed that cluster 6 was associated with EndMT (**Figure 7D**). Next, we investigated the dynamics of these clusters in galectin-7-overexpressing HAECs exposed to OSS, and we observed a significant reduction in the number of cells in cluster 6 following the overexpression of galectin-7 (**Figure 7E**). In addition, we identified DEGs for each cluster and performed KEGG pathway analysis and GO analysis. KEGG analysis revealed significant alterations in fluid shear stress, atherosclerosis, and the TGFβ signaling pathway in cluster 6 after overexpressing galectin-7 (**Figure 7F-G**). GO analysis revealed that cluster 6 had high levels of genes involved in cell migration, cellular response to fluid shear stress, and TGFβ binding (**Figure 7H**). A volcano plot revealed that the expression of the SRGN gene decreased most significantly after galectin-7 overexpression in cluster 6 (**Figure 7I**). The above results revealed that galectin-7 significantly downregulated SRGN expression in OSS-induced EndMT-associated cluster 6 cells.

### Endothelial galectin-7 inhibited disturbed flow-induced EndMT through the TGFβ/smad pathway in a SRGN-dependent manner

**Figure 8:**
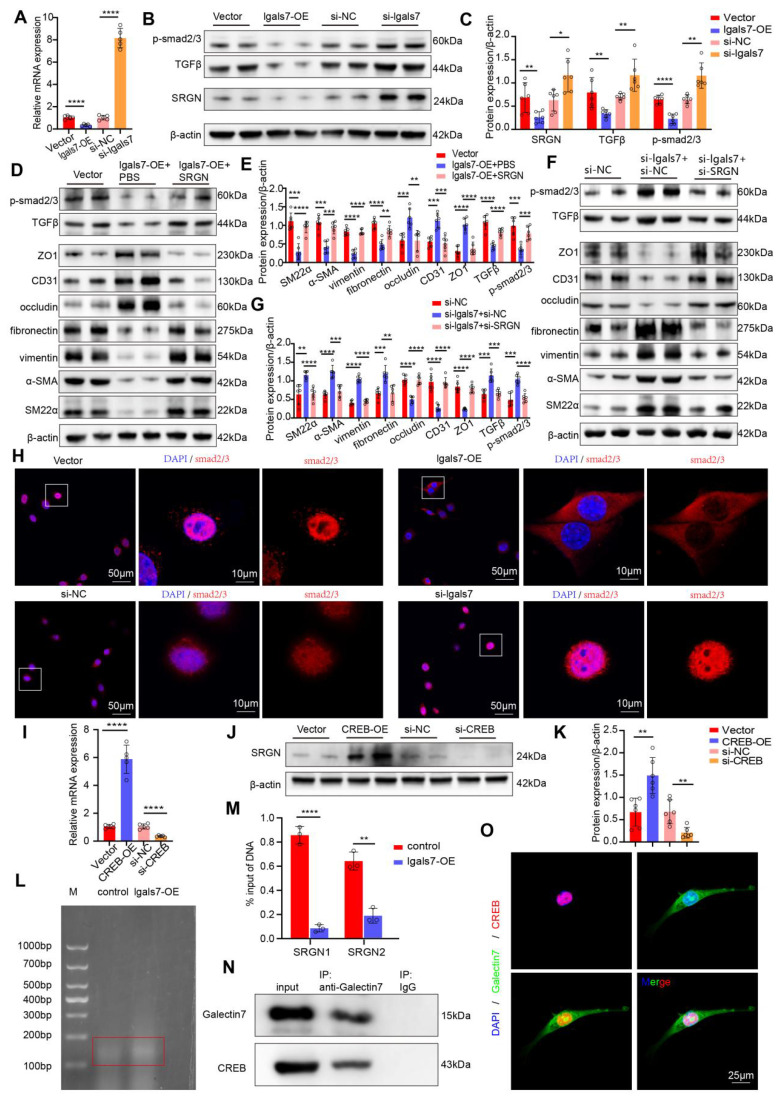
** Endothelial galectin-7 inhibits EndMT induced by disturbed flow through the TGFβ/smad pathway in an SRGN-dependent manner. A**, The relative PCR expression of SRGN in the 4 indicated groups (n = 5/group).** B-C**, Western blott and densitometric analysis of SRGN, TGFβ and p-smad2/3 in the 4 indicated groups (n = 6/group). **D-E**, Western blot and densitometric analyses of SM22α, α-SMA, vimentin, fibronectin, occludin, CD31, ZO1, TGFβ and p-smad2/3 in the 3 indicated groups (n = 6/group). **F-G**, Western blot and densitometric analysis of SM22α, α-SMA, vimentin, fibronectin, occludin, CD31, ZO1, TGFβ and p-smad2/3 in the 3 indicated groups (n = 6/group). **H**, Immunofluorescence staining of smad2/3 (red) in the 4 indicated groups. Nuclei were co-stained with DAPI (blue) (scale bars = 50μm and 10μm, n = 4/group). **I**, The relative PCR expression of SRGN in the 4 indicated groups (n = 5/group). **J-K**, Western blot and densitometric analysis of SRGN in the 4 indicated groups (n = 6/group). **L**, Detection of DNA fragmentation in treated cells. **M**, Quantitative ChIP-qPCR was used to assess the binding of CREB to the promoter regions of SRGN in HAECs. The percent input method was used to compare two groups (n = 3/group). **N**, HAECs were immunoprecipitated and then immunoblotted with antibodies against the indicated proteins (n = 3/group). **O**, Immunofluorescence staining of galectin-7 (green) and CREB (red). Nuclei were co-stained with DAPI (blue) (scale bars = 25μm, n = 4/group). One-way ANOVA with a post hoc Bonferroni's multiple comparisons test was performed for A, C, E, G, I and K. Unpaired Student's t-test was performed for M. *p < 0.05, **p < 0.01, ***p < 0.001, ****p < 0.0001.

As mentioned above, galectin-7 downregulates SRGN expression most significantly in cluster 6. Previous studies have demonstrated that the TGFβ pathway is downstream pathway of SRGN and plays a key role in the development of EndMT. On the basis of the above findings, we hypothesized that galectin-7 inhibits disturbed flow-induced EndMT through the SRGN/TGFβ/smad pathway. PCR and western blot results demonstrated that the overexpression of galectin-7 suppressed the expression of SRGN, whereas the knockdown of galectin-7 increased the expression of SRGN (**Figure 8A-C, Figure S13A-D**). Furthermore, we investigated whether galectin-7 regulates disturbed flow-induced EndMT through SRGN by performing rescue experiments and complementation assays. When HAECs were exposed to OSS* in vitro*, galectin-7 overexpression increased endothelial marker expression and decreased mesenchymal marker expression. These effects were reversed by SRGN overexpression (**Figure 8D-E**). Consistent with these findings, galectin-7 knockdown decreased the expression of endothelial markers and increased the expression of mesenchymal markers, whereas these effects were eliminated by SRGN knockdown (**Figure 8F-G**). In addition, western blotting results showed that galectin-7 overexpression inhibited TGFβ and phosphorylated smad2/3 expression, which was reversed by SRGN overexpression (**Figure 8D-E**). Galectin-7 knockdown promoted the expression of TGFβ and phosphorylated smad2/3, an effect that was abolished by SRGN knockdown, indicating that SRGN mediates the regulatory effect of galectin-7 on the TGFβ/smad pathway (**Figure 8F-G**). Moreover, immunofluorescence staining and western blotting results revealed that overexpression of galectin-7 inhibited smads nuclear translocation, whereas the knockdown of galectin-7 promoted smads nuclear translocation (**Figure 8H, Figure S14A-B**). However, western blotting revealed that overexpression of SRGN promoted the nuclear entry of smads, whereas the knockdown of SRGN inhibited nuclear entry of smads (**Figure S14C-D**). Notably, the effect of galectin-7 on smad nuclear translocation was reversed by SRGN (**Figure S14E-H**). The above results indicated that galectin-7 regulated disturbed flow-induced EndMT through the TGFβ/smad pathway in an SRGN-dependent manner.

Next, we explored the mechanism by which galectin-7 downregulated SRGN. Since galectin-7 regulated SRGN at the transcriptional level, we next attempted to identify the downstream transcription factor directly regulating SRGN transcription. Previous studies identified the binding sites of CREB in the promoter region of the human SRGN gene and confirmed that the interaction between CREB and the SRGN promoter region enhances the transcription of this gene. PCR and western blot results revealed that overexpression of CREB in OSS-exposed HAECs led to an increase in the expression of SRGN and that the knockdown of CREB resulted in a decrease in the expression of SRGN (**Figure 8I-K**). We next investigated whether galectin-7 affected CREB binding to the SRGN promoter region. The ChIP-qPCR results revealed that galectin-7 overexpression significantly reduced the ability of CREB to bind the SRGN promoter region (**Figure 8L-M**). In addition, Co-IP results revealed a direct interaction between galectin-7 and CREB (**Figure 8N**). Immunofluorescence staining revealed the co-localization of galectin-7 with CREB in the cell nucleus (**Figure 8O**). These results indicate that galectin-7 competitively binds to CREB, preventing CREB from binding to the SRGN promoter region and thereby repressing its transcription.

## Discussion

In the present study, we showed that the d-flow-induced EndMT drives AAA progression and further revealed that endothelial galectin-7 impedes the TGFβ/SMADs pathway in a SRGN-dependent manner in ECs, resulting in decreased EndMT and therefore constraining AAA formation and development. Our findings highlight that manipulating d-flow-induced EndMT might be a promising therapeutic strategy against AAA progression and that galectin-7 supplementation might be a potential avenue for AAA treatment.

The results of the current study provide the first insight into the effects of d-flow-induced EndMT on the formation and progression of AAA. As shown by our present data, the partial ligation of the suprarenal abdominal aorta aggravated EndMT, promoting the occurrence of AAA and increasing the aneurysm diameter. Moreover, we also found that treatment with an EndMT inhibitor significantly reduced the maximal aneurysm diameter and improved cumulative survival of AAA model mice, which further indicated the critical role of EndMT in the occurrence and development of AAA. D-flow promoted EndMT by inducing transcriptional phenotypes related to inflammation, hypoxia responses, glycolysis, and fatty acid synthesis^15^, ^30^-^32^. An increasing number of studies have shown that EndMT occurs in AAA^9^, ^33^, but a causal relationship has not been identified. We confirmed for the first time that the occurrence of EndMT is associated with blood flow patterns in AAA. More importantly, aortic aneurysms preferentially develop in the abdominal aorta, where the vessel wall is susceptible to d-flow^34^. In line with those observations, we compared the expression of endothelial and mesenchymal markers in the aortic arch, thoracic aorta, and abdominal aorta of mice with different blood flow patterns, and the results indicated that d-flow induced EndMT and L-flow had opposite effect on EndMT. Accordingly, our results revealed d-flow-induced EndMT contributes strongly to the etiology of AAA and might be an effective target for treating AAA caused by hypertension.

Another valuable finding of our study was that galectin-7 was shown to be a key mediator connecting d-flow and EMT and thus aortic aneurysm progression. Endothelial galectin-7was considered to be a flow-sensitive protein^26^ and we found that the expression of galectin-7 was closely related to blood flow shear stress. The present results indicate that endothelial galectin-7 expression significantly decreases in areas dominated by d-flow in the aortic arch and abdominal aorta and significantly increases in areas dominated by l-flow in the thoracic aorta. Moreover, we applied Ibidi pumps to create a d-flow environment *in vitro,* and the galectin-7 level, as determined by fluorescent staining and immunoblotting, were consistent with the *in vivo* trends. More importantly, our study also confirmed that galectin-7, which is limited by d-flow, is associated with the occurrence of EndMT, further leading to the occurrence of AAA. Our *in vivo* and *in vitro* data show that knockdown or overexpression of galectin-7 leads to progression or regression of aortic aneurysms and that this change in phenotype is mediated primarily by EndMT. Stress-related molecular changes have important potential for the treatment of vascular disorders^35^. The present findings reveal the significant role of galectin-7 in stress responses and provide a target for improvements in fluid dynamics to intervene in AAA and other stress-endothelial disfunction-related disorders. Notably, in our present study, we verified that galectin-7 is localized in the intima of human abdominal aorta and that its level is decreased in human AAA, indicating the clinical significance of galectin-7 for AAA. Therefore, these results suggest that galectin-7, an indispensable molecule that links d-flow and EndMT, suppress AAA progression by inhibiting the EndMT, suggesting that it may be a new target for aortic aneurysm intervention.

We also elucidated the mechanism by which galectin-7 functions in d-flow-induced EndMT. Our single-cell RNA sequencing results revealed that overexpression of galectin-7 reduced the proportion of the EndMT-related gene cluster and that the altered expressions of SRGN was the most prominent in this cluster. Previous studies have shown that SRGN is a novel shear stress-responsive gene and is expressed mainly in ECs^36^. We performed rescue experiments and found that galectin-7 inhibited d-flow-induced EndMT by downregulating SRGN expression. Many studies have shown that the regulation of molecular transcription level is achieved primarily through the influence of transcription factors^37^. Co-IP and ChIP experiments revealed that galectin-7 can competitively bind the transcription factor CREB to transcriptionally inhibit SRGN. The SRGN/TGFβ axis plays a critical role in the development of EndMT and thus participated in various pathological processes, such as atherosclerosis and tumor^38^, ^39^. Our data revealed that the overexpression of galectin-7 decreased the expression of TGFβ and phosphorylated SMAD2/3 and inhibited the nuclear translocation of SMADs, effect that were reversed by overexpression of SRGN. Taken together, these results confirm that galectin-7 suppresses d-flow-induced EndMT by inhibiting the TGFβ/SMADs pathway in an SRGN-dependent manner.

Several limitations of the present study should be acknowledged and need to be further addressed. First, we revealed that endothelial galectin-7 is a key regulator of disturbed flow-induced EndMT in AAA. However, whether other endothelial galectins plays a specific role in regulating d-flow-induced EndMT in AAA remains to be elucidated in future studies. Secondly, it should be noted that the embryonic origin of the thoracic aorta and the abdominal aorta is different, which may lead to differences in the behavior of endothelial cells between them. Future research will conduct separation and comparison at more precise anatomical locations to comprehensively depict the endothelial function among different segments of the aorta. Finally, this study focused on d-flow-induced EndMT as the primary cause of the formation and progression of AAA; further studies need to focus on whether a reduction in d-flow after blood pressure control delays AAA progression by inhibiting the EMT process.

In conclusion, our study demonstrated that d-flow-induced EndMT promotes the formation and progression of AAA and that endothelial galectin-7 suppresses d-flow-induced EndMT to constrain AAA progression through the inhibition of the transcription of SRGN and, subsequently, the TGFβ/SMADs pathway. Therefore, manipulating the galectin-7-mediated inhibition of d-flow-induced EndMT may be a novel treatment approach for AAA.

## Supplementary Material

Supplementary figures and tables.

## References

[1] Zuin M, Aggarwal R, Bikdeli B, Kirksey L, Hussain MA, Bilato MJ (2024). Abdominal Aortic Aneurysm-Attributed Mortality in the United States. Journal of the American College of Cardiology.

[2] Butnariu LI, Russu G, Luca AC, Sandu C, Trandafir LM, Vasiliu I (2024). Identification of Genetic Variants Associated with Hereditary Thoracic Aortic Diseases (HTADs) Using Next Generation Sequencing (NGS) Technology and Genotype-Phenotype Correlations. International journal of molecular sciences.

[3] Yang P, Liu H, Wang S, Xiao X, Jiang L, Le S (2024). PIEZO1 attenuates Marfan syndrome aneurysm development through TGF-β signaling pathway inhibition via TGFBR2. European heart journal.

[4] Ma Y, Li D, Cui F, Wang J, Tang L, Yang Y (2024). Air pollutants, genetic susceptibility, and abdominal aortic aneurysm risk: a prospective study. European heart journal.

[5] Hibino M, Otaki Y, Kobeissi E, Pan H, Hibino H, Taddese H (2022). Blood Pressure, Hypertension, and the Risk of Aortic Dissection Incidence and Mortality: Results From the J-SCH Study, the UK Biobank Study, and a Meta-Analysis of Cohort Studies. Circulation.

[6] Bossone E, Eagle KA (2021). Epidemiology and management of aortic disease: aortic aneurysms and acute aortic syndromes. Nature reviews Cardiology.

[7] Klopf J, Zagrapan B, Brandau A, Lechenauer P, Candussi CJ, Rossi P (2024). Circulating monocyte populations as biomarker for abdominal aortic aneurysms: a single-center retrospective cohort study. Front Immunol.

[8] Czaja B, Závodszky G, Azizi Tarksalooyeh V, Hoekstra AG (2018). Cell-resolved blood flow simulations of saccular aneurysms: effects of pulsatility and aspect ratio. J R Soc Interface.

[9] Terriaca S, Scioli MG, Bertoldo F, Pisano C, Nardi P, Balistreri CR (2024). miRNA-Driven Regulation of Endothelial-to-Mesenchymal Transition Differs among Thoracic Aortic Aneurysms. Cells.

[10] Bellien J, Iacob M, Richard V, Wils J, Le Cam-Duchez V, Joannidès R (2021). Evidence for wall shear stress-dependent t-PA release in human conduit arteries: role of endothelial factors and impact of high blood pressure. Hypertension research: official journal of the Japanese Society of Hypertension.

[11] Kaneko N, Mashiko T, Namba K, Tateshima S, Watanabe E, Kawai K (2018). A patient-specific intracranial aneurysm model with endothelial lining: a novel *in vitro* approach to bridge the gap between biology and flow dynamics. J Neurointerv Surg.

[12] Na JT, Hu SY, Xue CD, Wang YX, Chen KJ, Li YJ (2021). A microfluidic system for precisely reproducing physiological blood pressure and wall shear stress to endothelial cells. The Analyst.

[13] Morgan B, Murali AR, Preston G, Sima YA, Marcelo Chamorro LA, Bourantas C (2023). A physics-based machine learning technique rapidly reconstructs the wall-shear stress and pressure fields in coronary arteries. Frontiers in cardiovascular medicine.

[14] Liang G, Wang S, Shao J, Jin YJ, Xu L, Yan Y (2022). Tenascin-X Mediates Flow-Induced Suppression of EndMT and Atherosclerosis. Circ Res.

[15] Chen LJ, Li JY, Nguyen P, He M, Chen ZB, Subramaniam S (2024). Single-cell RNA sequencing unveils unique transcriptomic signatures of endothelial cells and role of ENO1 in response to disturbed flow. Proceedings of the National Academy of Sciences of the United States of America.

[16] Pang ZD, Sun X, Bai RY, Han MZ, Zhang YJ, Wu W (2023). YAP-galectin-3 signaling mediates endothelial dysfunction in angiotensin II-induced hypertension in mice. Cellular and molecular life sciences: CMLS.

[17] Souchak J, Mohammed NBB, Lau LS, Dimitroff CJ (2024). The role of galectins in mediating the adhesion of circulating cells to vascular endothelium. Frontiers in immunology.

[18] Sotoudeheian MJ, Mirahmadi SM, Pirhayati M, Azarbad R, Nematollahi S, Taghizadeh M (2024). Understanding the Role of Galectin-1 in Heart Failure: A Comprehensive Narrative Review. Current cardiology reviews.

[19] Camarda ND, Ibarrola J, Biwer LA, Jaffe IZ (2024). Mineralocorticoid Receptors in Vascular Smooth Muscle: Blood Pressure and Beyond. Hypertension (Dallas, Tex: 1979).

[20] Chen HL, Lo CH, Huang CC, Lu MP, Hu PY, Chen CS (2021). Galectin-7 downregulation in lesional keratinocytes contributes to enhanced IL-17A signaling and skin pathology in psoriasis. The Journal of clinical investigation.

[21] Sun L, Liu R, Wu ZJ, Liu ZY, Wan AH, Yan S (2024). Galectin-7 Induction by EHMT2 Inhibition Enhances Immunity in Microsatellite Stability Colorectal Cancer. Gastroenterology.

[22] Menkhorst E, Zhou W, Santos L, Zhang JG, St-Pierre Y, Young MJ (2022). Galectin-7 dysregulates renin-angiotensin-aldosterone and NADPH oxide synthase pathways in preeclampsia. Pregnancy hypertension.

[23] Pinto NA, Abba MC, Laporte L, Pérez Sáez JM, Blidner AG, Torres NI (2023). Galectin-7 reprograms skin carcinogenesis by fostering innate immune evasive programs. Cell death and differentiation.

[24] Sun X, Shen W, Li Z, Zhang W (2022). CCCTC-binding factor transcriptionally regulates Galectin-7 and activates the JNK/STAT3 axis to aggravate bronchial epithelial cell injury. Pediatric pulmonology.

[25] Alvandi Z, Bischoff J (2021). Endothelial-Mesenchymal Transition in Cardiovascular Disease. Arteriosclerosis, thrombosis, and vascular biology.

[26] Sewgobind NV, Albers S, Pieters RJ (2021). Functions and Inhibition of Galectin-7, an Emerging Target in Cellular Pathophysiology. Biomolecules.

[27] He X, Li X, Han Y, Chen G, Xu T, Cai D (2022). CircRNA Chordc1 protects mice from abdominal aortic aneurysm by contributing to the phenotype and growth of vascular smooth muscle cells. Mol Ther Nucleic Acids.

[28] Zhao G, Chang Z, Zhao Y, Guo Y, Lu H, Liang W (2021). KLF11 protects against abdominal aortic aneurysm through inhibition of endothelial cell dysfunction. JCI Insight.

[29] Li X, Guo S, Xu T, He X, Sun Y, Chen X (2020). Therapeutic ultrasound combined with microbubbles improves atherosclerotic plaque stability by selectively destroying the intraplaque neovasculature. Theranostics.

[30] Andueza A, Kumar S, Kim J, Kang DW, Mumme HL, Perez JI (2020). Endothelial Reprogramming by Disturbed Flow Revealed by Single-Cell RNA and Chromatin Accessibility Study. Cell reports.

[31] Campinho P, Lamperti P, Boselli F, Vilfan A, Vermot J (2020). Blood Flow Limits Endothelial Cell Extrusion in the Zebrafish Dorsal Aorta. Cell reports.

[32] Zhao R, Yi Y, Liu H, Xu J, Chen S, Wu D (2024). RHOF promotes Snail1 lactylation by enhancing PKM2-mediated glycolysis to induce pancreatic cancer cell endothelial-mesenchymal transition. Cancer & metabolism.

[33] Terriaca S, Scioli MG, Pisano C, Ruvolo G, Ferlosio A, Orlandi A (2023). miR-632 Induces DNAJB6 Inhibition Stimulating Endothelial-to-Mesenchymal Transition and Fibrosis in Marfan Syndrome Aortopathy. International journal of molecular sciences.

[34] Wei Y, Jiang H, Li F, Chai C, Xu Y, Xing M (2024). Extravascular administration of IGF1R antagonists protects against aortic aneurysm in rodent and porcine models. Science translational medicine.

[35] Iring A, Jin YJ, Albarrán-Juárez J, Siragusa M, Wang S, Dancs PT (2019). Shear stress-induced endothelial adrenomedullin signaling regulates vascular tone and blood pressure. The Journal of clinical investigation.

[36] Ma Q, Gu W, Li T, Zhang K, Cui Y, Qu K (2020). SRGN, a new identified shear-stress-responsive gene in endothelial cells. Molecular and cellular biochemistry.

[37] Alim I, Caulfield JT, Chen Y, Swarup V, Geschwind DH, Ivanova E (2019). Selenium Drives a Transcriptional Adaptive Program to Block Ferroptosis and Treat Stroke. Cell.

[38] Tellez-Gabriel M, Tekpli X, Reine TM, Hegge B, Nielsen SR, Chen M (2022). Serglycin Is Involved in TGF-β Induced Epithelial-Mesenchymal Transition and Is Highly Expressed by Immune Cells in Breast Cancer Tissue. Frontiers in oncology.

[39] Shao X, Hou X, Zhang X, Zhang R, Zhu R, Qi H (2023). Integrated single-cell RNA-seq analysis reveals the vital cell types and dynamic development signature of atherosclerosis. Frontiers in physiology.

