# Supplementary material for: Galectin7 attenuates abdominal aortic aneurysm progression by resisting disturbed flow induced endothelial-to-mesenchymal transition
Source: Theranostics. 2026 Jan 8;16(7):3665–84. doi: 10.7150/thno.117785 (PMC12846751; doi:10.7150/thno.117785)
Supplement: Supplementary file 1 — Supplementary figures and tables. [file thnov16p3665s1.pdf]

Supplemental figure legends

Supplemental Figure 1. EndMT in HAECs was induced by OSS *in vitro*.

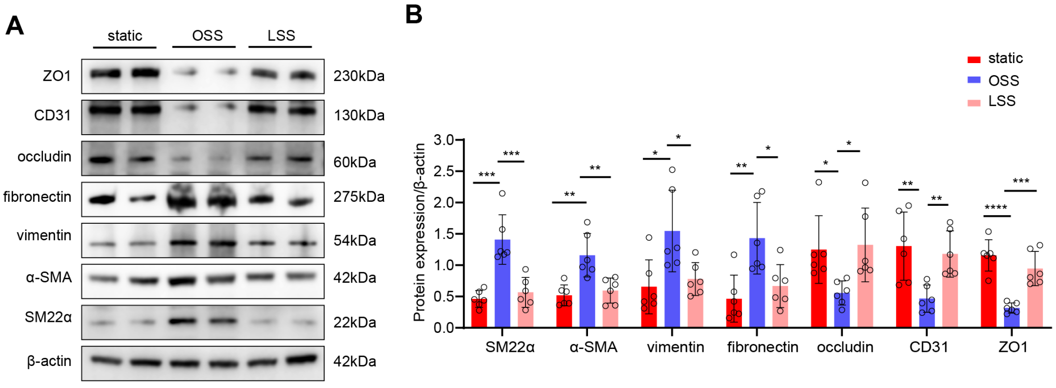

A-B, Western blotting and densitometric analysis of SM22α, α-SMA, vimentin, fibronectin, occludin, CD31 and ZO1 protein expression levels in HAECs in the 3 indicated groups (n = 6/group). One-way ANOVA with a post hoc Bonferroni's multiple comparisons test was performed for B. *p < 0.05, **p < 0.01, ***p < 0.001, ****p < 0.0001.

Supplemental Figure 2. D-flow-induced EndMT is present in mouse Ang II-induced AAA model.

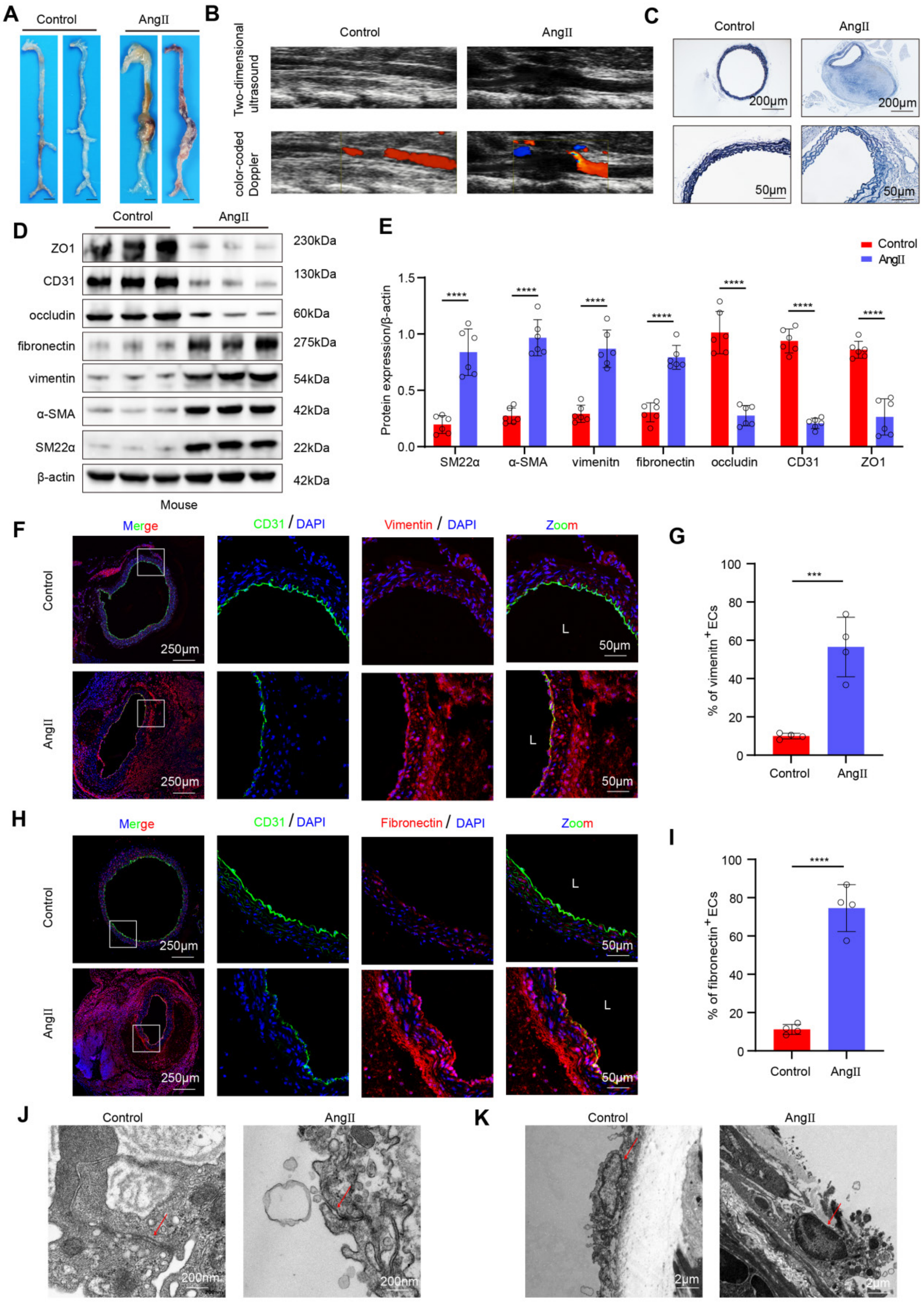

A, Representative macroscopic photographs of the suprarenal abdominal aorta in male ApoE$^{-/-}$ mice at 28 d after saline or Ang II infusion. **B**, Two-dimensional ultrasound and color-coded Doppler imaging of aortas after saline and Ang II infusion in male ApoE$^{-/-}$ mice. **C**, Representative EVG staining images of the mouse aortas (scale bars = 200μm and 50μm, n = 8/group). **D-E**, Western blotting and densitometric analysis of SM22α, α-SMA, vimentin, fibronectin, occludin, CD31 and ZO1 protein expression levels in the endothelium of control aortas and Ang II-induced AAA (n = 6/group). **F-I**, Double immunofluorescence staining for CD31 (green) and vimentin (red) or fibronectin (red). Nuclei were co-stained with DAPI (blue) (scale bars = 250μm and 50μm, n = 4/group). **J**, Representative transmission electron microscopy images of control and AngII-induced AAA endothelial TJ ultrastructures (scale bars = 200nm). **K**, Representative images of transmission electron microscopy of control and AngII-induced AAA endothelial cell morphology (scale bars = 2μm). Unpaired Student’s t-test was performed for E, G and I. $*p < 0.05$, $**p < 0.01$, $***p < 0.001$, $****p < 0.0001$.

Supplemental Figure 3. D-flow-induced EndMT is present in mouse PPE-induced AAA model.

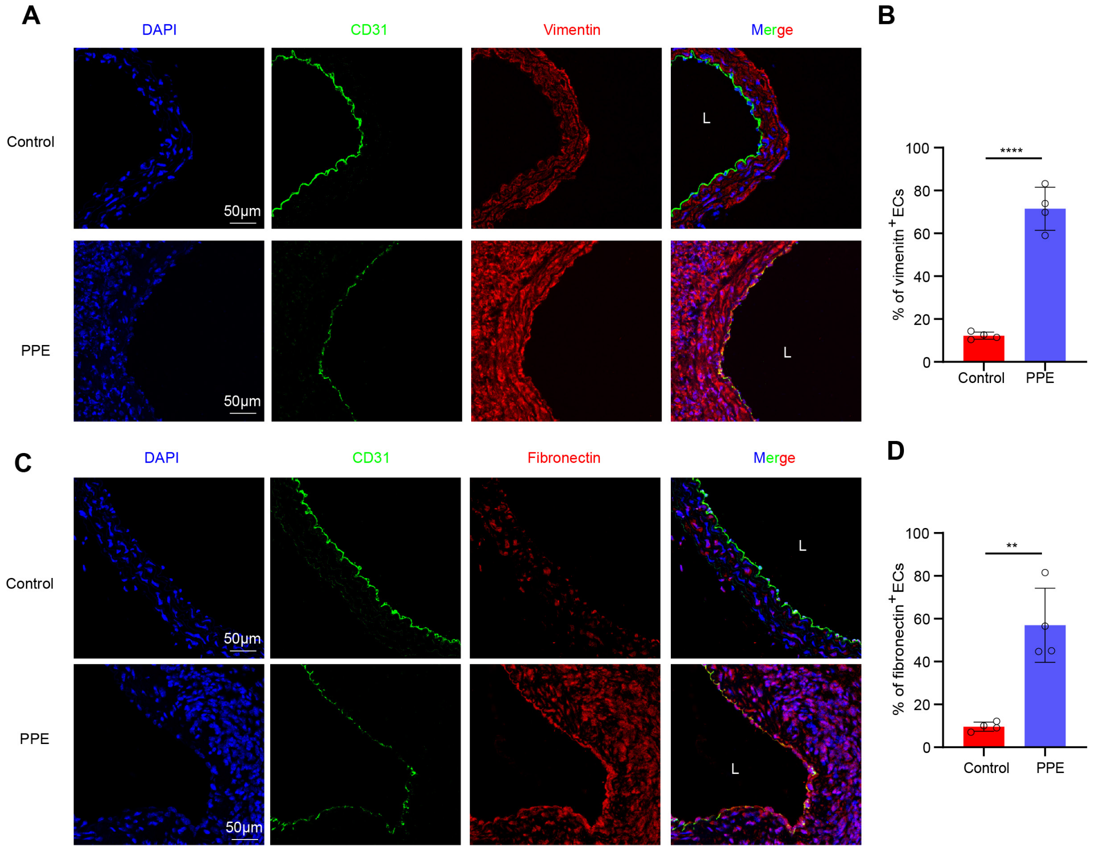

A-D, Representative immunofluorescence staining images and densitometric analysis for CD31 (green) and vimentin (red) or fibronectin (red). Nuclei were co-stained with DAPI (blue) (scale bars = 50μm, n = 4/group). Unpaired Student' s t-test was performed for B and D. *p < 0.05, **p < 0.01, ***p < 0.001, ****p < 0.0001.

Supplemental Figure 4. Genotypic identification of the endothelial lineage-tracing mouse model.

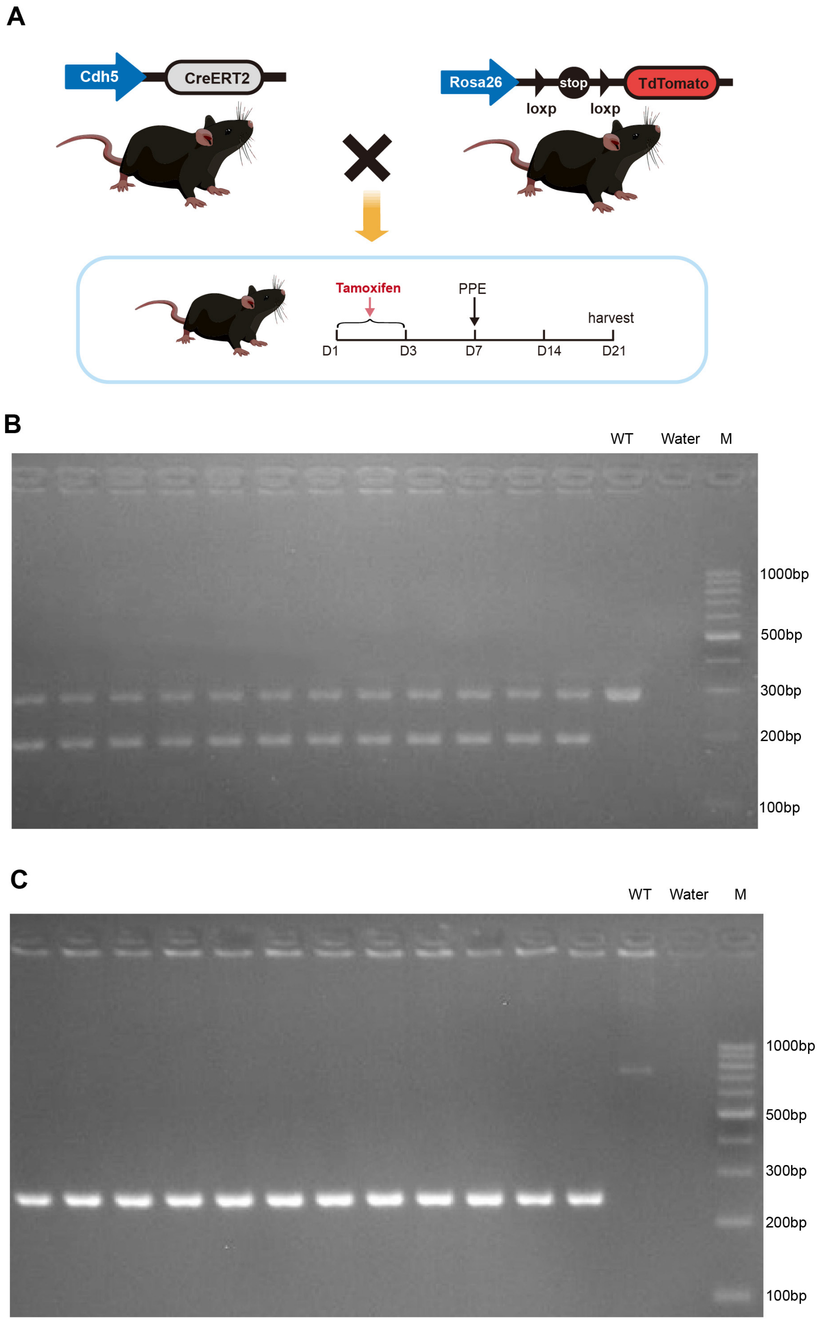

A, Schematic of the endothelial lineage-tracing strategy. **B**, PCR analysis for the identification of the tdTomato reporter allele. **C**, PCR analysis for the detection of the Cdh5-CreERT2 transgene.

Supplemental Figure 5. The activation of Renin-Angiotensin System (RAS) is not the primary driver for AAA aggravation after partial ligation of the suprarenal abdominal aorta.

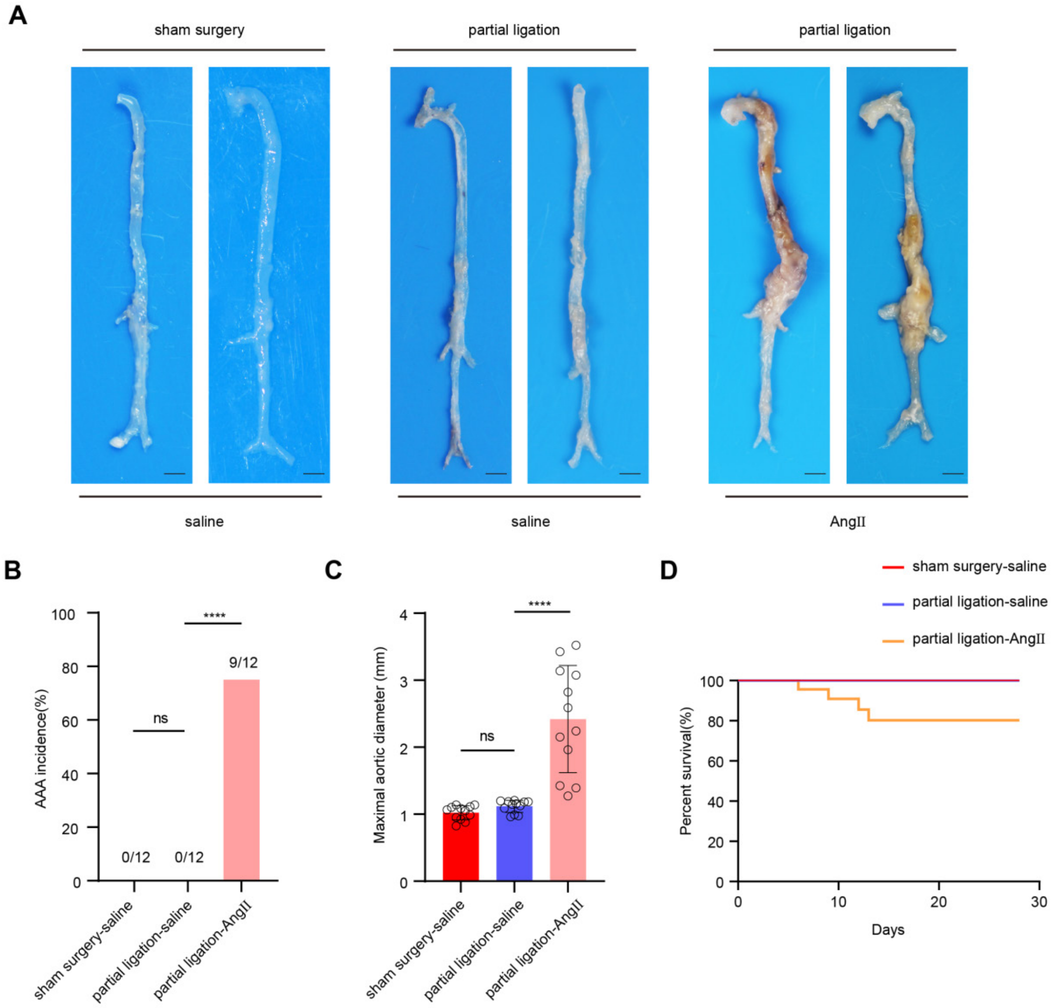

A, Representative macroscopic photographs of the suprarenal abdominal aorta in male ApoE$^{-/-}$ mice at 28 d after saline or Ang II infusion in 3 indicated groups. **B**, The AAA incidence in the 3 indicated groups (n = 12/group). **C**, The maximal abdominal aortic diameter of 3 indicated groups (n = 12/group). **D**, The survival curves of AngII-induced AAA in the 3 indicated groups (n = 12/group). Fisher's exact test was performed for B. One-way ANOVA with a post hoc Bonferroni's multiple comparisons test was performed for C. *$p < 0.05$, **$p < 0.01$, ***$p < 0.001$, ****$p < 0.0001$.

Supplemental Figure 6. QPCR results for lgals7 in human AAA samples.

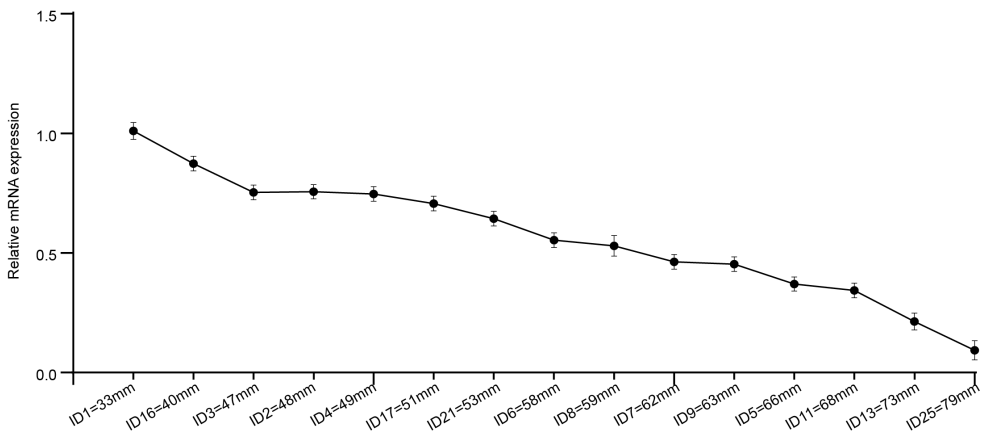

The relative PCR expression of galectin-7 in human AAA samples.

Supplemental Figure 7. EC-specific overexpression of galectin-7 prevents PPE-

induced AAA progression.

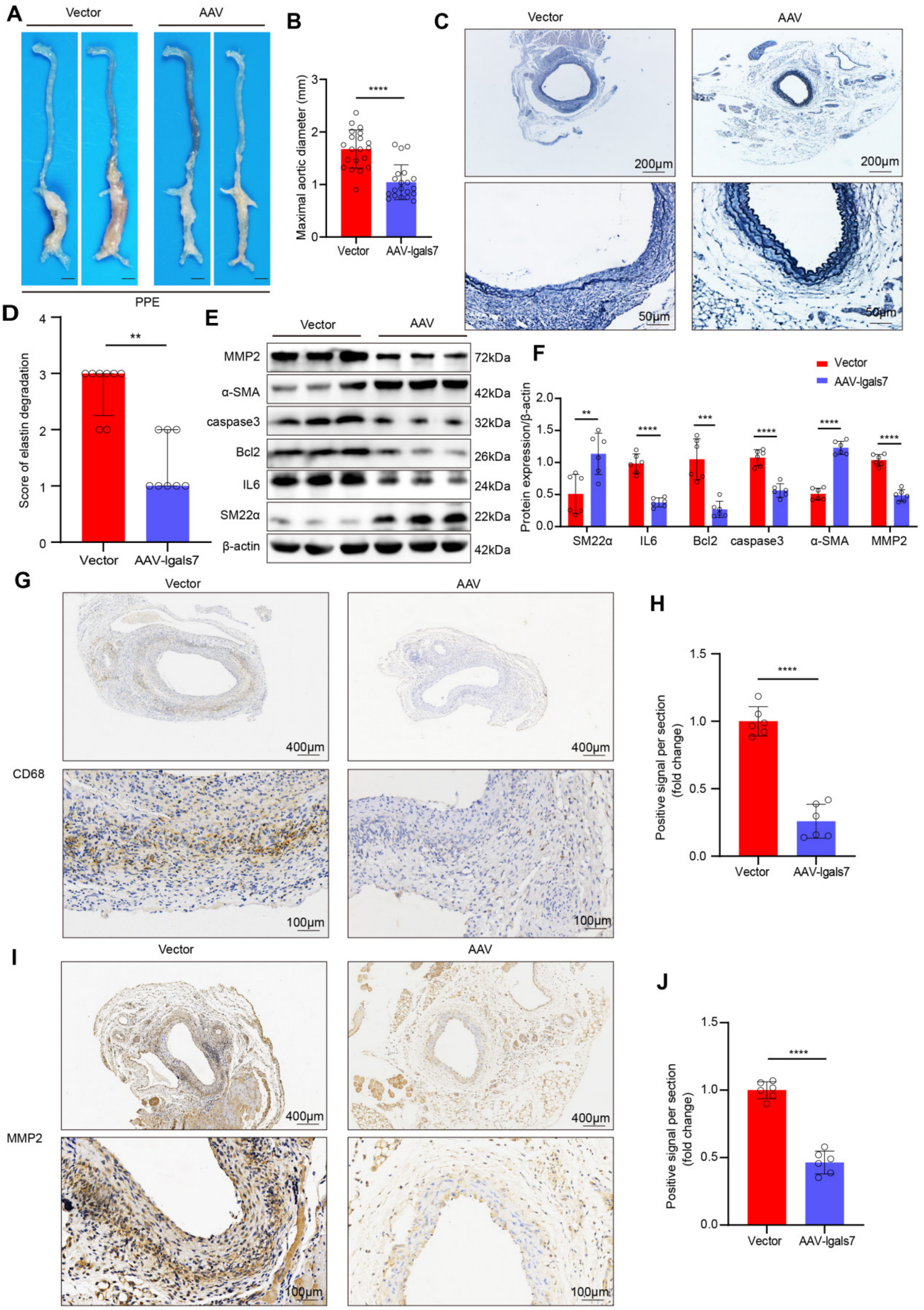

A, Representative macroscopic photographs of infrarenal abdominal aorta from PPE-induced AAA in the two indicated groups. **B**, The maximal abdominal aortic diameter of PPE-induced AAA in the two indicated groups (n = 20/group). **C-D**, Representative

EVG staining images and elastin degradation scores in the two indicated groups (scale bars = 200μm and 50μm, n = 8/group). **E-F**, Western blotting and densitometric analysis of SM22α, IL6, Bcl2, caspase3, α-SMA and MMP2 protein expression levels of PPE-induced AAA in the two indicated groups (n = 6/group). **G-J**, Representative immunohistochemical staining of CD68 (G) and MMP2 (I) and corresponding densitometric analysis (scale bars = 400μm and 100μm, n = 6/group). Unpaired Student's t-test was performed for B, F, H and J. Mann-Whitney test was performed for D. $*p < 0.05$, $**p < 0.01$, $***p < 0.001$, $****p < 0.0001$.

Supplemental Figure 8. EC-specific knockdown of galectin-7 promotes PPE-induced AAA progression.

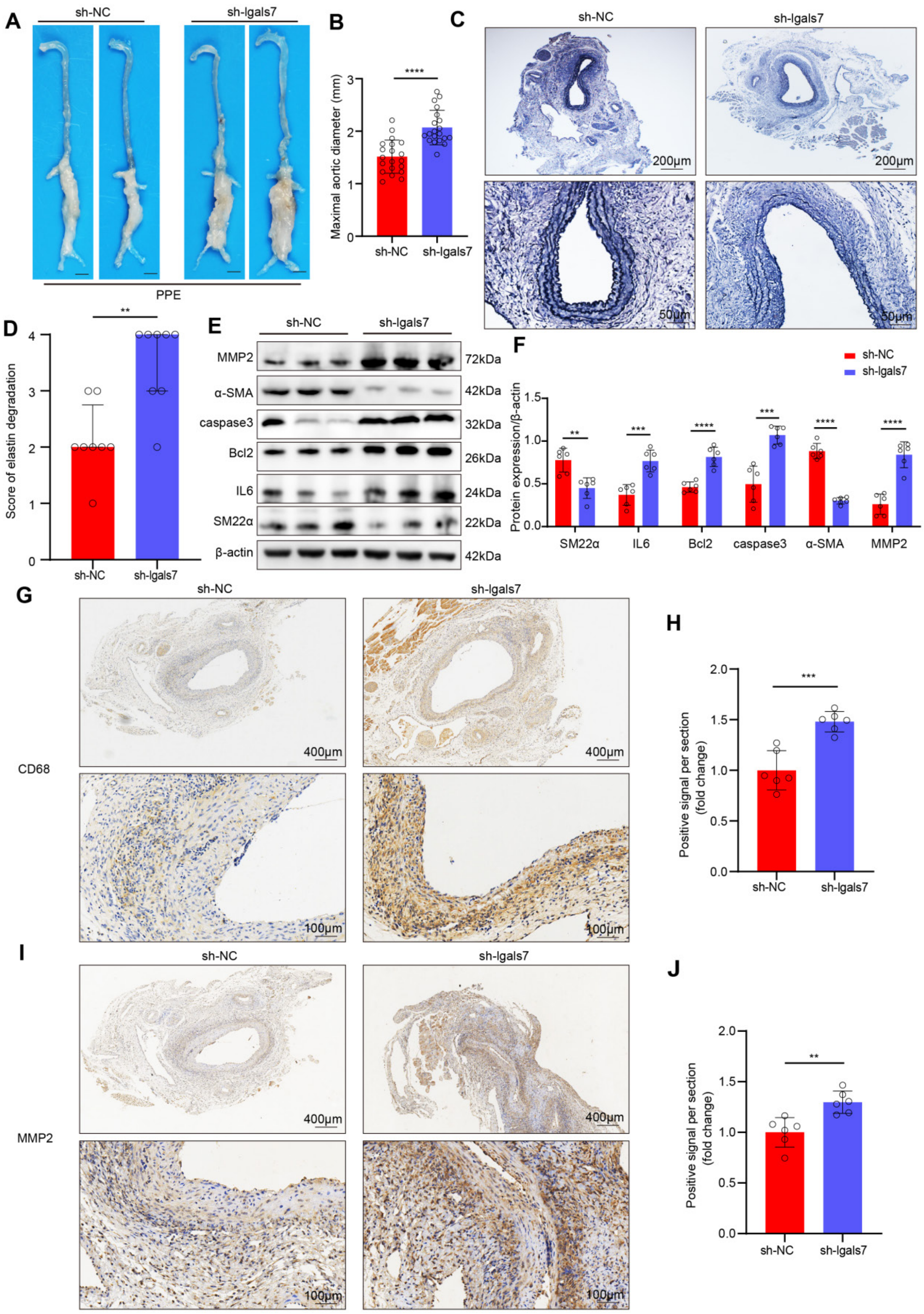

A, Representative macroscopic photographs of infrarenal abdominal aorta from PPE-induced AAA in the two indicated groups. **B**, The maximal abdominal aortic diameter of PPE-induced AAA in sh-NC group and sh-lgals7 group. (n = 20/group). **C-D**, Representative EVG staining images and elastin degradation scores in the two indicated

groups (scale bars = 200μm and 50μm, n = 8/group). **E-F**, Western blotting and densitometric analysis of SM22α, IL6, Bcl2, caspase3, α-SMA and MMP2 protein expression levels of PPE-induced AAA in sh-NC group and sh-lgals7 group. (n = 6/group). **G-J**, Representative immunohistochemical staining of CD68 (G) and MMP2 (I) and corresponding densitometric analysis (scale bars = 400μm and 100μm, n = 6/group). Unpaired Student's t-test was performed for B, F, H and J. Mann-Whitney test was performed for D. *p < 0.05, **p < 0.01, ***p < 0.001, ****p < 0.0001.

Supplemental Figure 9. EC-specific overexpression of galectin-7 inhibits disturbed flow-induced EndMT and EC-specific knockdown of galectin-7 promotes disturbed flow-induced EndMT in Ang II-induced AAA.

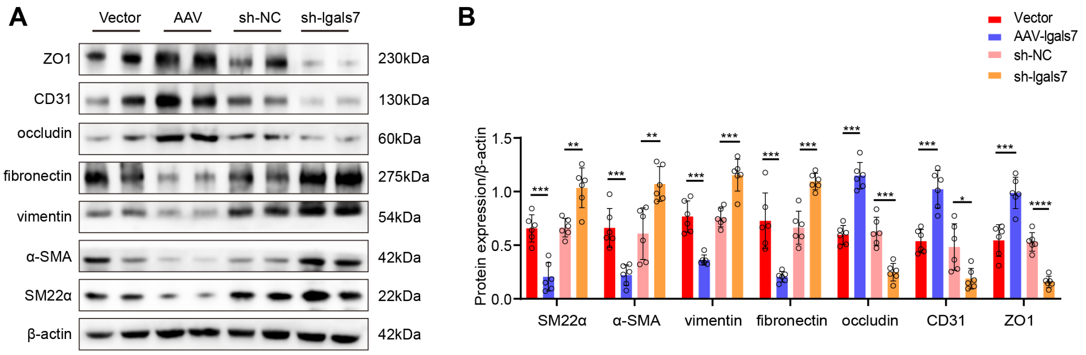

A-B, Western blotting and densitometric analysis of SM22α, α-SMA, vimentin, fibronectin, occludin, CD31 and ZO1 protein expression levels in the endothelium of AngII-induced AAA in the 4 indicated groups (n = 6/group). One-way ANOVA with a post hoc Bonferroni's multiple comparisons test was performed for B. *p < 0.05, **p < 0.01, ***p < 0.001, ****p < 0.0001.

Supplemental Figure 10. EC-specific overexpression of galectin-7 inhibits disturbed flow-induced EndMT and EC-specific knockdown of galectin-7 promotes disturbed flow-induced EndMT in PPE-induced AAA.

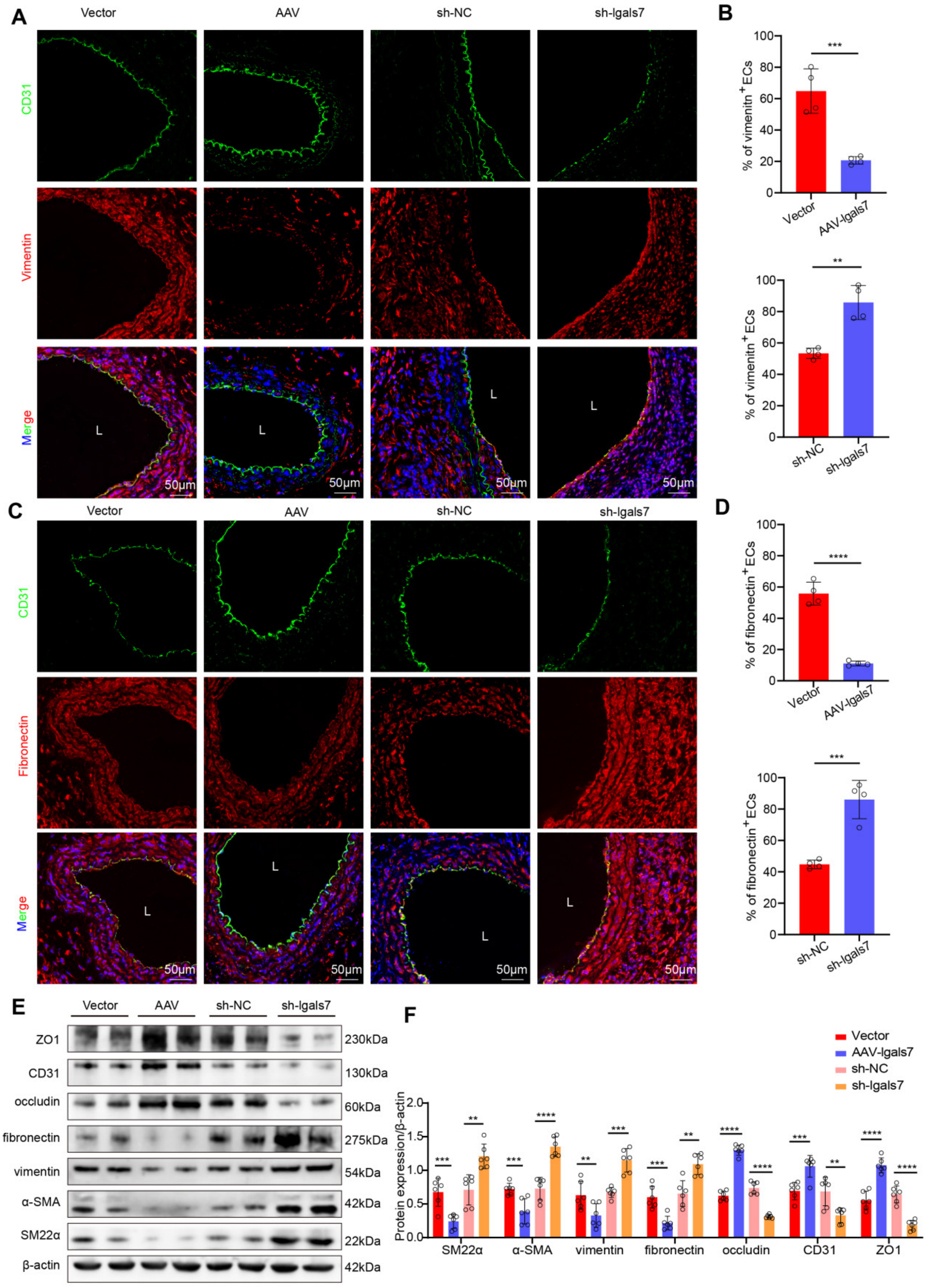

A-B, Representative immunofluorescence staining images and densitometric analysis for CD31 (green) and vimentin (red) in the 4 indicated groups. Nuclei were co-stained with DAPI (blue) (scale bars = 50μm, n = 4/group). **C-D**, Representative immunofluorescence staining images and densitometric analysis for CD31 (green) and

fibronectin (red) in the 4 indicated groups. Nuclei were co-stained with DAPI (blue) (scale bars = 50μm, n = 4/group). **E-F**, Western blotting and densitometric analysis of SM22α, α-SMA, vimentin, fibronectin, occludin, CD31 and ZO1 protein expression levels in the endothelium of PPE-induced AAA in the 4 indicated groups (n = 6/group). Unpaired Student’ s t-test was performed for B and D. One-way ANOVA with a post hoc Bonferroni's multiple comparisons test was performed for F. *p < 0.05, **p < 0.01, ***p < 0.001, ****p < 0.0001.

Supplemental Figure 11. Overexpression of galectin-7 inhibits OSS-induced EndMT and knockdown of galectin-7 promotes OSS-induced EndMT in HAECs.

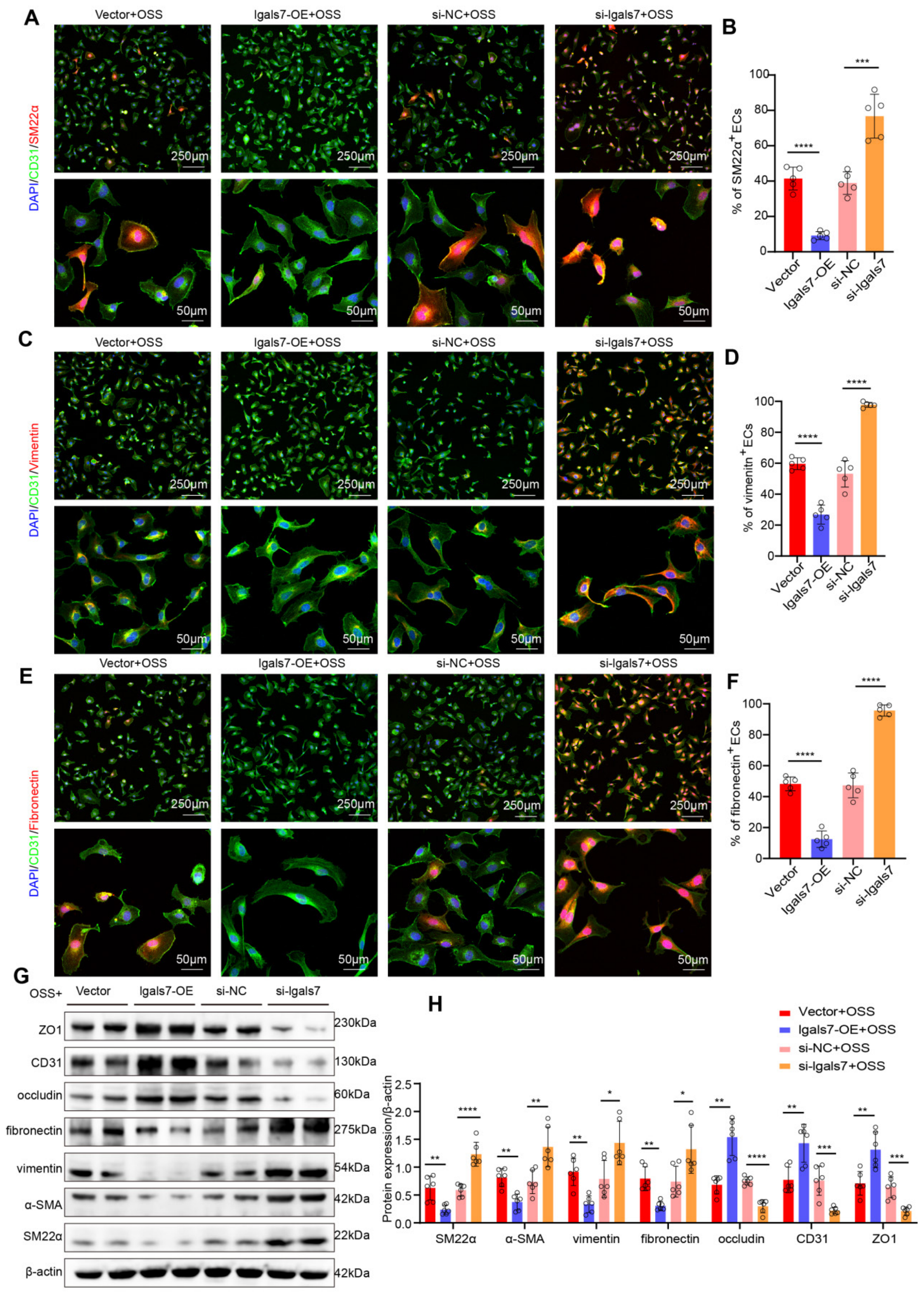

A-B, Representative immunofluorescence staining images and densitometric analysis for CD31 (green) and SM22α (red) in HAECs in the 4 indicated groups. Nuclei were co-stained with DAPI (blue) (scale bars = 250μm and 50μm, n = 5/group). **C-D**, Representative immunofluorescence staining images and densitometric analysis for

CD31 (green) and vimentin (red) in HAECs in the 4 indicated groups. Nuclei were co-stained with DAPI (blue) (scale bars = 250μm and 50μm, n = 5/group). **E-F**, Representative immunofluorescence staining images and densitometric analysis for CD31 (green) and fibronectin (red) in HAECs in the 4 indicated groups. Nuclei were co-stained with DAPI (blue) (scale bars = 250μm and 50μm, n = 5/group). **G-H**, Western blotting and densitometric analysis of SM22α, α-SMA, vimentin, fibronectin, occludin, CD31 and ZO1 protein expression levels in HAECs in the 4 indicated groups (n = 6/group). One-way ANOVA with a post hoc Bonferroni's multiple comparisons test was performed for B, D, F and H. *p < 0.05, **p < 0.01, ***p < 0.001, ****p < 0.0001.

Supplemental Figure12. Endothelial galectin-7 suppresses Ang II-Induced AAA progression by inhibiting d-flow-induced EndMT.

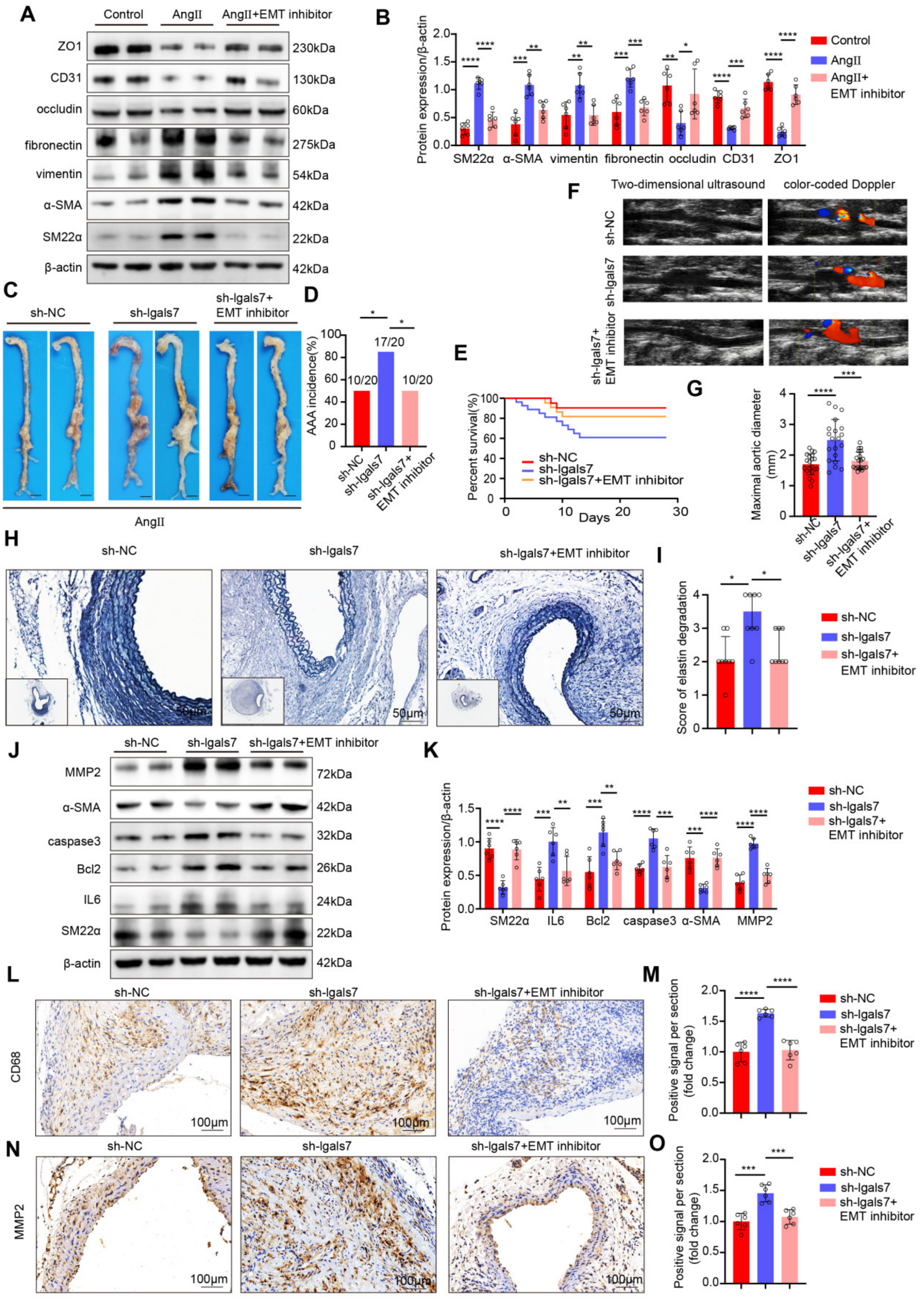

A-B, Western blotting of protein expression levels and densitometric analysis of aortic endothelial SM22α, α-SMA, vimentin, fibronectin, occludin, CD31 and ZO1 in the 3 indicated groups (n = 6/group). **C**, Representative macroscopic photographs of suprarenal AAA in the 3 indicated groups after AngII induction. **D**, The AngII-induced

AAA incidence in the 3 indicated groups (n = 20/group). **E**, The survival curves of AngII-induced AAA in the 3 indicated groups (n=20/group). **F**, Two-dimensional ultrasound and color-coded Doppler imaging of AngII-induced AAA in the 3 indicated groups. **G**, The maximal abdominal aortic diameter of AngII-induced AAA (n = 20/group). **H-I**, Representative staining with elastin and the elastin degradation scores in the 3 indicated groups (scale bars = 200μm and 50μm, n = 8/group). **J-K**, Western blotting of protein expression levels and densitometric analysis of aortic SM22α, IL6, Bcl2, caspase3, α-SMA and MMP2 in the AngII-induced AAA (n = 6/group). **L-O**, Representative immunohistochemical staining of CD68 (L) and MMP2 (N) and corresponding densitometric analysis (scale bars = 100μm, n = 6/group). One-way ANOVA with a post hoc Bonferroni's multiple comparisons test was performed for B, G, K, M and O. Fisher's exact test was performed for D. Kruskall-Wallis with Dunn's multiple comparisons test was performed for I. $*p < 0.05$, $**p < 0.01$, $***p < 0.001$, $****p < 0.0001$.

Supplemental Figure 13. The protein expression levels of SRGN in Ang Ⅱ and PPE induced AAA were compared among the four groups.

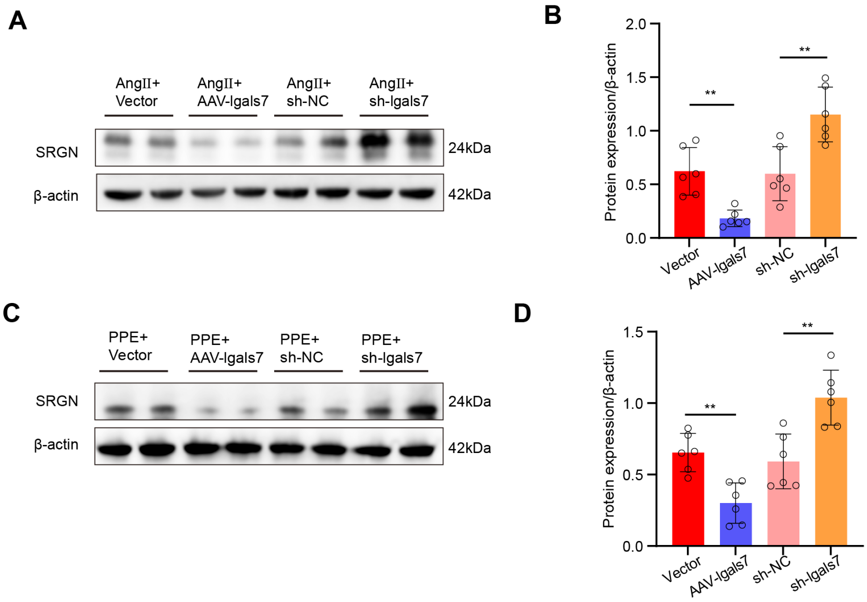

A-B, Western blotting and densitometric analysis of SRGN protein expression levels in AngII-induced AAA in the 4 indicated groups (n = 6/group). **C-D**, Western blotting and densitometric analysis of SRGN protein expression levels in PPE-induced AAA in the 4 indicated groups (n = 6/group). One-way ANOVA with a post hoc Bonferroni's multiple comparisons test was performed for B and D. $*p < 0.05$, $**p < 0.01$, $***p < 0.001$, $****p < 0.0001$.

Supplemental Figure 14. Galectin-7 inhibits smads nuclear translocation by down-regulating SRGN.

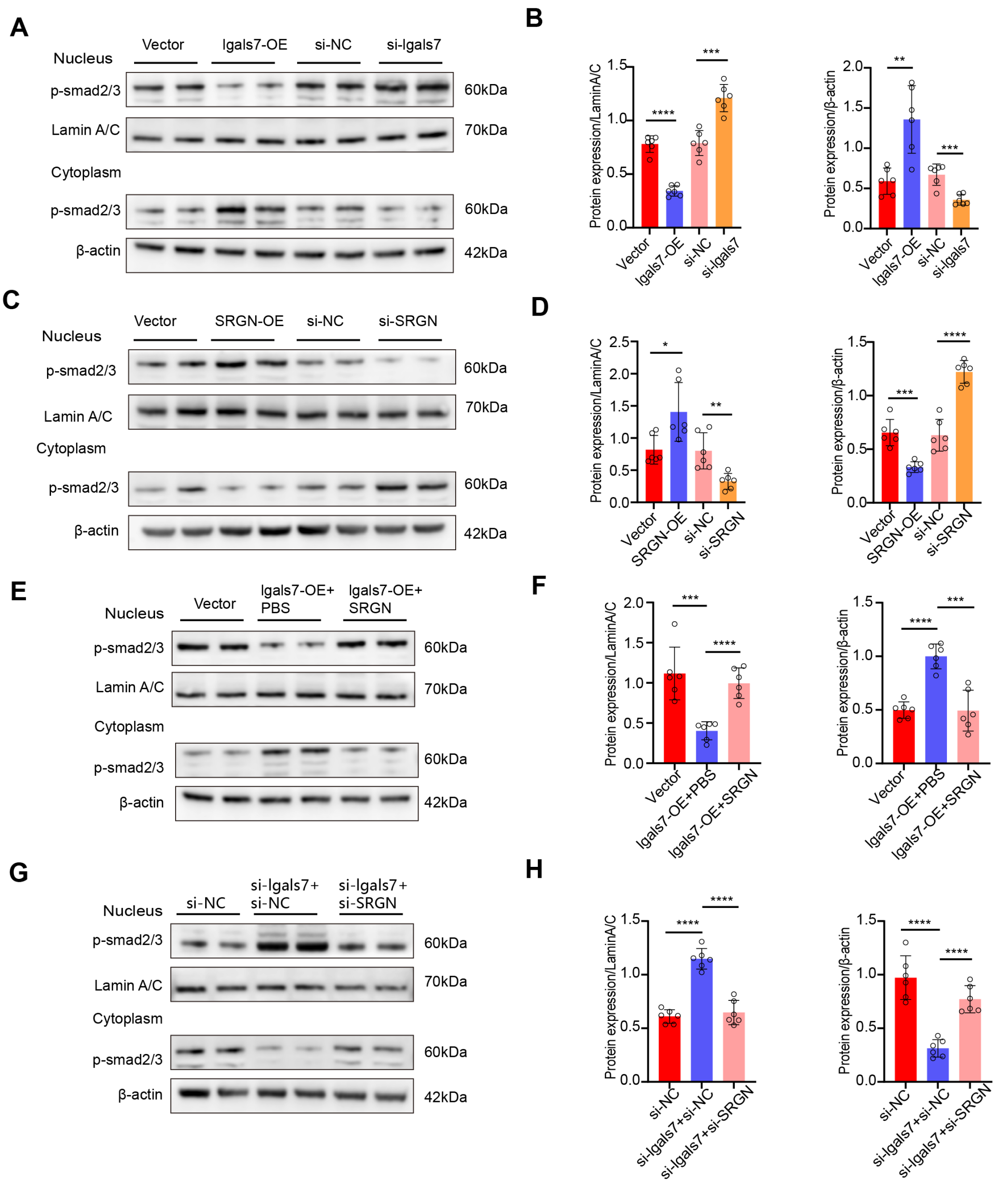

A-B, Western blotting of protein expression levels and densitometric analysis of p-smad2/3 from nuclear and cytoplasmic proteins in the 4 indicated groups (n = 6/group). **C-D**, Western blotting of protein expression levels and densitometric analysis of p-smad2/3 from nuclear and cytoplasmic proteins in the 4 indicated groups (n = 6/group). **E-F**, Western blotting of protein expression levels and densitometric analysis of p-smad2/3 from nuclear and cytoplasmic proteins in the 3 indicated groups (n = 6/group). **G-H**, Western blotting of protein expression levels and densitometric analysis of p-smad2/3 from nuclear and cytoplasmic proteins in the 3 indicated groups (n = 6/group). One-way ANOVA with a post hoc Bonferroni's multiple comparisons test was

performed for B, D, F and H. *p < 0.05, **p < 0.01, ***p < 0.001, ****p < 0.0001.

Supplemental Figure 15. Confirmation of the viral transfection site and efficiency in the suprarenal aortas of C57BL/6J mice.

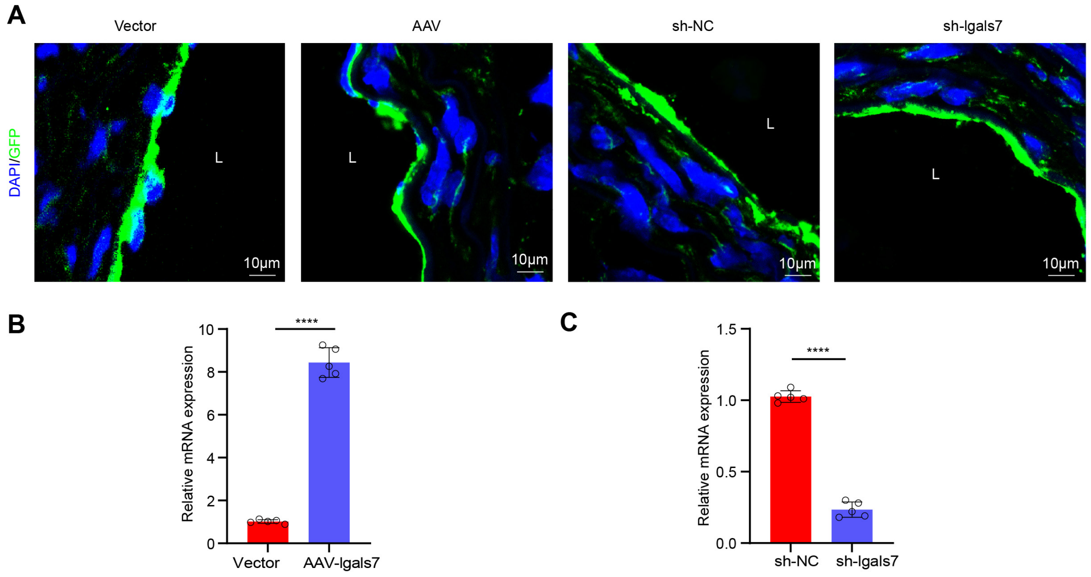

A, Representative immunofluorescence staining of virus in the mouse aortas in the 4 indicated groups (scale bars = 10μm). **B**, The relative PCR expression demonstrates the overexpression efficiency of galectin-7 (n = 5/group). **C**, The relative PCR expression demonstrates the knockdown efficiency of galectin-7 (n = 5/group). Unpaired Student' s t-test was performed for B-C. *p < 0.05, **p < 0.01, ***p < 0.001, ****p < 0.0001.

Supplemental Figure 16. Immunofluorescence confirmation of high purity of isolated endothelial cells.

A

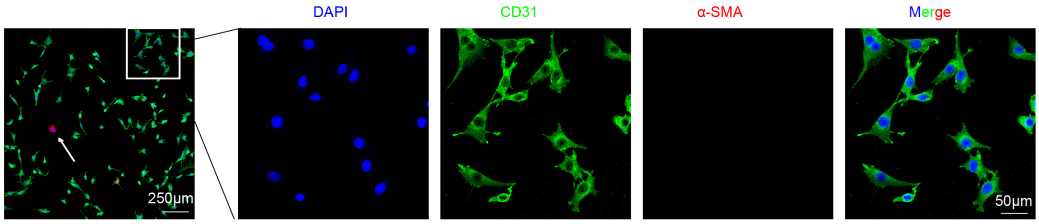

A, Representative immunofluorescence staining images for CD31 (green) and α-SMA (red) in isolated endothelial cells. Nuclei were co-stained with DAPI (blue) (scale bars = 250μm and 50μm).

Supplemental materials

Table S1: Patient information for human aortic samples (n=25)

Patient ID	gender	age	smoking status	aortic diameter (mm)	hyper lipidemia	hyper tensive	coronary artery disease
ID1	Female	60	No	33	No	No	No
ID2	Male	53	Yes	48	No	Yes	No
ID3	Male	58	Yes	47	No	Yes	No
ID4	Male	53	Yes	49	Yes	No	No
ID5	Female	73	No	66	No	Yes	No
ID6	Male	48	No	58	No	Yes	No
ID7	Male	47	Yes	62	No	Yes	No
ID8	Male	58	No	59	No	No	No
ID9	Male	59	No	63	No	Yes	Yes
ID10	Female	65	No	44	No	No	No
ID11	Male	49	No	68	No	Yes	No
ID12	Male	53	Yes	60	No	Yes	No
ID13	Male	78	No	73	No	Yes	No
ID14	Male	56	No	45	No	No	No
ID15	Male	80	Yes	78	Yes	Yes	Yes
ID16	Male	74	Yes	40	Yes	Yes	No
ID17	Male	87	Yes	51	No	Yes	No
ID18	Male	70	No	72	No	No	No
ID19	Male	66	Yes	63	No	No	Yes
ID20	Male	67	Yes	55	No	No	No
ID21	Male	69	No	53	No	Yes	No
ID22	Male	67	Yes	62	No	Yes	No
ID23	Male	75	No	63	No	Yes	No
ID24	Male	88	No	58	No	Yes	Yes
ID25	Male	66	Yes	79	Yes	Yes	No

Table S2: Sequence of primers used in Quantitative real-time PCR

Primer	Sequence(5' → 3')
M-GAPDH-F	TGTCCGTCGTGGATCTGAC
M-GAPDH-R	CCTGCTTCACCACCTTCTTG
M-SM22α-F	AAGCAGATGGAACAGGTGGC
M-SM22α-R	CACAGCCAAACTGCCCAAAG
M-α-SMA-F	CTACGAACTGCCTGACGGG
M-α-SMA-R	GCTGTTATAGGTGGTTTCGTGG
M-Vimentin-F	GGACCAGCTAACCAACGACA
M-Vimentin-R	AAGGTCAAGACGTGCCAGAG
M-fibronectin-F	ATCGCATTGGGGATCAGTGG

M-fibronectin-R	CACTGGTCAATGGGGTCACAC
M-occludin-F	TCTTTCCTTAGGCGACAGCG
M-occludin-R	AGATAAGCGAACCTGCCGAG
M-CD31-F	GAGCCTCACCAAGAGAACGG
M-CD31-R	TCTCGCAATCCAGGAATCGG
M-ZO1-F	AGACGCCCGAGGGTGTAG
M-ZO1-R	TGGGACAAAAGTCCGGGAAG
M-lgals7-F	TTCCACGTGAACCTGCTGT
M-lgals7-R	GAAAGTGGTGGTACTGTGCG
H-GAPDH-F	ACATCGCTCAGACACCATG
H-GAPDH-R	TGTAGTTGAGGTCAATGAAGGG
H-lgals7-F	CAGACGACGGCTTCAAGG
H-lgals7-R	AAGATCCTCACGGAGTCCAG
H-SRGN-F	AGTTTCACTTCACGAGCTTGG
H-SRGN-R	TAGGAAATGTAGCAGGGTGCTC
**Primers for ChIP-PCR**	
SRGN promoter-F1	GGAGTCCAGTACAGTTTC
SRGN promoter-R1	CCCAGAACACACGTCAC
SRGN promoter-F2	GGACCAAAGATGACATCTG
SRGN promoter-R2	CCTCACATCAGCCAGAAGTG
GAPDH-CHIP-F	CAGGAGGAGCAGAGAGCG
GAPDH-CHIP-R	GGACTGGCTGAGCCTGG

Table S3: Antibodies for immunohistochemistry analysis

Name	Source	Catalog #
anti-CD68	proteintech	28058-1-AP
anti-MMP2	proteintech	10373-2-AP
anti-Galectin7	Abcam	ab206435

Table S4: Antibodies for immunofluorescent analysis

Name	Source	Catalog #
anti-Galectin7	Abcam	ab206435
anti-Galectin7	R&D Systems	MAB13392
anti-CD31	R&D Systems	AF3628
anti-SM22α	proteintech	10493-1-AP
anti-Vimentin	proteintech	10366-1-AP
anti-fibronectin	proteintech	15613-1-AP
anti-smad2/3	Invitrogen	PA5-99539
anti-CREB	Cell Signaling Technology	9197

Table S5: Antibodies for Co-Immunoprecipitation(Co-IP)

Name	Source	Catalog #
anti-Galectin7	Abcam	ab206435

Table S6: Antibodies for Chromatin Immunoprecipitation(CHIP)

Name	Source	Catalog #
anti-CREB	Cell Signaling Technology	9197

Table S7: Antibodies for western blots

Name	Source	Catalog #
anti-SM22α	proteintech	10493-1-AP
anti-α-SMA	proteintech	14395-1-AP
anti-Vimenitn	proteintech	10366-1-AP
anti-fibronectin	proteintech	15613-1-AP
anti-occludin	proteintech	27260-1-AP
anti-CD31	proteintech	28083-1-AP
anti-ZO1	proteintech	21773-1-AP
anti-β-actin	Bioss	bs-0061R
anti-IL6	proteintech	26404-1-AP
anti-Bcl2	proteintech	26593-1-AP
anti-caspase3	proteintech	19677-1-AP
anti-MMP2	proteintech	10373-2-AP
anti-Galectin7	Invitrogen	MA5-43812
anti-Galectin7	Abcam	ab206435
anti-TGFβ	proteintech	21898-1-AP
anti-p-smad2/3	Cell Signaling Technology	8828
anti-SRGN	ABclonal Technology	A25426
anti-CREB	Cell Signaling Technology	9197
anti-LaminA/C	proteintech	10298-1-AP
